# Antidepressant and Antipsychotic Drugs Reduce Viral Infection by SARS-CoV-2 and Fluoxetine Shows Antiviral Activity Against the Novel Variants *in vitro*

**DOI:** 10.3389/fphar.2021.755600

**Published:** 2022-01-19

**Authors:** Senem Merve Fred, Suvi Kuivanen, Hasan Ugurlu, Plinio Cabrera Casarotto, Lev Levanov, Kalle Saksela, Olli Vapalahti, Eero Castrén

**Affiliations:** ^1^ Neuroscience Center–HiLIFE, University of Helsinki, Helsinki, Finland; ^2^ Department of Virology, University of Helsinki, Helsinki, Finland; ^3^ Department of Veterinary Biosciences, University of Helsinki, Helsinki, Finland; ^4^ HUS Diagnostic Center, HUSLAB, Clinical Microbiology, University of Helsinki and Helsinki University Hospital, Helsinki, Finland

**Keywords:** antidepressants (AD), fluoxetine, SARS-CoV-2, alpha variant, beta variant, delta variant

## Abstract

Repurposing of currently available drugs is a valuable strategy to tackle the consequences of COVID-19. Recently, several studies have investigated the effect of psychoactive drugs on SARS-CoV-2 in cell culture models as well as in clinical practice. Our aim was to expand these studies and test some of these compounds against newly emerged variants. Several antidepressants and antipsychotic drugs with different primary mechanisms of action were tested in ACE2/TMPRSS2-expressing human embryonic kidney cells against the infection by SARS-CoV-2 spike protein-dependent pseudoviruses. Some of these compounds were also tested in human lung epithelial cell line, Calu-1, against the first wave (B.1) lineage of SARS-CoV-2 and the variants of concern, B.1.1.7, B.1.351, and B.1.617.2. Several clinically used antidepressants, including fluoxetine, citalopram, reboxetine, imipramine, as well as antipsychotic compounds chlorpromazine, flupenthixol, and pimozide inhibited the infection by pseudotyped viruses with minimal effects on cell viability. The antiviral action of several of these drugs was verified in Calu-1 cells against the B.1 lineage of SARS-CoV-2. By contrast, the anticonvulsant carbamazepine, and novel antidepressants ketamine, known as anesthetic at high doses, and its derivatives as well as MAO and phosphodiesterase inhibitors phenelzine and rolipram, respectively, showed no activity in the pseudovirus model. Furthermore, fluoxetine remained effective against pseudoviruses with common receptor binding domain mutations, N501Y, K417N, and E484K, as well as B.1.1.7 (alpha), B.1.351 (beta), and B.1.617.2 (delta) variants of SARS-CoV-2. Our study confirms previous data and extends information on the repurposing of these drugs to counteract SARS-CoV-2 infection including different variants of concern, however, extensive clinical studies must be performed to confirm our *in vitro* findings.

## Introduction

Coronaviruses, members of the enveloped RNA virus family *Coronaviridae* (Lai & Cavanagh, 1997)*,* are known to infect multiple species ranging from birds to mammals (To et al., 2013). In humans, in addition to four coronaviruses causing common colds, high level of pathogenicity of viruses from this family, such as severe acute respiratory syndrome coronavirus (SARS-CoV) and Middle East respiratory syndrome coronavirus (MERS-CoV), were observed in epidemics that emerged in 2003 and 2012, respectively (Tang et al., 2015). The pandemic that started in December 2019 in Wuhan, China, caused by SARS-CoV-2 infection, and the dramatic increase in the number of infected people and death due to COVID-19, has shifted the efforts of scientists towards investigating this virus more closely (F. Wu et al., 2020; Zhou et al., 2020). Autopsies of patients who died from COVID-19 have reported the spread of virus to lungs, kidneys, liver, heart, brain, and blood (Puelles et al., 2020). Taken together, severity of cases and high infectivity of the virus requires more attention and effort towards controlling the global epidemic and developing treatment options.

The spike protein (S protein) localized to the viral envelope has been annotated as one of the critical parts of this group of viruses because of its role in attachment and fusion to host target cells (Li et al., 2005). Angiotensin-converting enzyme 2 (ACE2) located on the surface of the target cells is recognized by S protein of SARS-CoV and SARS-CoV-2 (Li et al., 2003; Walls et al., 2020; Wang et al., 2020; Hoffmann, et al., 2020b). A distinct location, the receptor-binding domain (RBD), in S protein is important for binding to ACE2 (Li et al., 2005; Wan et al., 2020). ACE2-binding alone may allow the viral entry to target cells, however, proteolytic processing of S protein by transmembrane protease/serine subfamily member 2 (TMPRSS2) has been shown to enhance viral entry (Shulla et al., 2011; Hoffmann, et al., 2020b). Recent data on SARS-CoV-2 also suggest that the S protein cleavage by furin proprotein convertase (Hoffmann, et a., 2020a; Johnson et al., 2021) and binding of the C-end of the S1 to the cellular receptor neuropilin-1 can also increase infectivity (Cantuti-Castelvetri et al., 2020; Daly et al., 2020). Lysosomal enzyme Cathepsin L has been shown to regulate priming of SARS-CoV-2 S protein for the entry of viral RNA genome into the host cytoplasm (Ou et al., 2020). Although many key players facilitating viral entry and the routes of infection have been identified since the emergence of COVID-19, effective drugs that can alleviate or eliminate the viral infection remain to be found.

A number of SARS-CoV-2 variants of concern have been identified, including B.1.1.7 lineage (alpha) (Rambaut, 2020), B.1.351 lineage (beta) (Tegally et al., 2020), B.1.1.28 lineage and the descendent P1 lineage (gamma) (Voloch et al., 2020; Faria et al., 2021; PAOLA, 2021), and one of the recent ones, B.1.617 and its sub-linage B.1.617.2 (delta) (Cherian et al., 2021; Mlcochova et al., 2021). Several common and critical mutations in the RBD of S protein have been detected in these variants which can affect the ACE2 affinity (Khateeb et al., 2021). A common RBD mutation, N501Y, is shared by alpha, beta, and gamma, while K417N and E484K are found in beta and gamma (Khateeb et al., 2021). The delta variant contains a non-RBD mutation, D614G, shared by alpha and gamma variants, as well as an RBD mutation, E484Q, which is similar to E484K in other variants (Khateeb et al., 2021). These mutations detected in the RBD domain of spike have been associated with antibody neutralization (Gu et al., 2020; Starr et al., 2020; Alenquer et al., 2021; Nelson et al., 2021; Wibmer et al., 2021; Greaney, et al., 2021a; Greaney, et al., 2021b). Moreover, they have also put the currently available vaccines and vaccine candidates under dispute (Fontanet et al., 2021).

The strategy of drug repurposing has been used as a method of searching novel treatments for virus-related diseases (Dyall et al., 2014; Mercorelli et al., 2018; Serra et al., 2021). A pre-clinical study has shown that the antidepressant drugs, such as sertraline, paroxetine, and clomipramine, can reduce the *Zaire* Ebola virus (EBOV) entry to target cells (Johansen et al., 2015). Recently, a number of studies presented evidence supporting the effects of antidepressant drugs and related psychoactive drugs as antiviral compounds against SARS-CoV-2 (Carpinteiro et al., 2020; Drayman et al., 2020; Schloer et al., 2020; Yang et al., 2020; Zimniak et al., 2021). In line with preclinical studies, the treatment of COVID-19 patients with fluoxetine, escitalopram, and venlafaxine for 20 days has been found to reduce the risk of intubation or death by COVID-19 (Hoertel, et al., 2021b). Treatment of COVID-19 patients with fluvoxamine for 2 weeks was also effective to decrease the development of clinical deterioration (Lenze et al., 2020). A follow up randomized controlled trial which was performed with higher number of patients has reached to the same conclusion about fluvoxamine that this compound helps with the reduction of hospitalization risk due to COVID-19 (Reis et al., 2021). Another study reported that the prevalence of COVID-19 was higher in health care professionals compared to patients in psychiatric ward of a hospital in Paris, which prompted the authors to suggest that the consumption of chlorpromazine protects against COVID-19 (Plaze et al., 2020a; Plaze et al., 2020b).

In the present study, we addressed if the psychoactive drugs can be used to reduce SARS-CoV-2 infection of host cells *in vitro*. We show that pharmacologically diverse antidepressant drugs, as well as several antipsychotics were able to reduce the infection by pseudotyped viruses harboring SARS-CoV-2 S protein. Treatment of human lung epithelial cell line Calu-1 infected with the B.1 lineage of SARS-CoV-2 with these drugs was also successful in reducing the amount of infectious virus. Moreover, infection by pseudotyped viruses carrying N501Y, K417N, or E484K single point mutations or triple mutation (N501Y/K417N/E484K) in the spike protein was shown to be reduced by fluoxetine. Fluoxetine was also effective against the variants of SARS-CoV-2, B.1.1.7 (alpha), B.1.351 (beta), and B.1.617.2 (delta) in Calu-1 cells.

## Materials and Methods

### Ethical Statement

Not applicable, all the experiments were performed using cells.

### Drugs

Fluoxetine (#H6995, Bosche Scientific), citalopram (#C505000, Toronto Research Chemicals), paroxetine (#2141, Tocris), fluvoxamine (#1033), venlafaxine (#2917, Tocris), reboxetine (#1982, Tocris), imipramine (#I7379-5G, Sigma–Aldrich), clomipramine (#C7291**,** Sigma–Aldrich), desipramine (#3067, Tocris), phenelzine (#P6777, Sigma–Aldrich), rolipram (#R6520, Sigma–Aldrich), ketamine (#3131, Tocris), 2R, 6R-Hydroxynorketamine and 2S, 6S-Hydroxynorketamine (#6094 and #6095, respectively, Tocris), carbamazepine (#4098, Tocris), chlorpromazine (#C8138, Sigma–Aldrich), flupenthixol (#4057, Tocris), and pimozide (#0937, Tocris) were investigated in this study. The compounds were dissolved in dimethyl sulfoxide (DMSO) that was also used as a vehicle in all the experiments. Concentrations tested (Table 1) were selected based on earlier *in vitro* studies from our research group (Fred et al., 2019; Casarotto et al., 2021) and other laboratories (Johansen et al., 2015).

**TABLE 1:** Psychoactive drugs implicated in our study and the doses used for the treatment of HEK 293T-ACE2-TMPRSS2 and Calu-1 cells.

Category of drugs tested	Name of drugs	Dose (µM)
Selective serotonin reuptake inhibitors (SSRIs)	Fluoxetine	0.01–20
	Citalopram	0.01–50
	Paroxetine	0.01–20
	Fluvoxamine	0.01–50
Selective norepinephrine reuptake inhibitor (NRI)	Reboxetine	0.1–50
Serotonin - norepinephrine reuptake inhibitor (SNRI)	Venlafaxine	0.01–50
Tricyclic antidepressants	Clomipramine	0.01–10
	Imipramine	0.01–50
	Desipramine	0.01–20
Rapid-acting antidepressants	Ketamine	0.01–50
	2R, 6R-Hydroxynorketamine (2R, 6R-HNK)	
	2S, 6S-Hydroxynorketamine (2S, 6S-HNK)	
Phosphodiesterase-4 inhibitor	Rolipram	0.01–50
Monoamine oxidase inhibitor	Phenelzine	0.01–50
Antipsychotic	Chlorpromazine	0.01–5
	Flupenthixol	0.01–10
	Pimozide	1–10
Anticonvulsant	Carbamazepine	0.1–50

### Cell Lines

TMPRSS2 (NM_001135099.1) and ACE2 (AB046569.1) cDNAs were sequentially transfected into HEK293T cells using lentiviral vectors. The positive colonies were selected by blasticidin or puromycin resistance for TMPRSS2 and ACE2, respectively. The viral vectors were generated by transfecting HEK293T cells using polyethylenimine with the packaging vector pCMV-dR8.91 and the vesicular stomatitis virus (VSV-G) envelope expression vector pMD2.G (#12259, Addgene) with either pLenti6.3/V5-DEST-TMPRSS2 (from UH Biomedicum Functional Genomic Unit) or pWPI-puro [modified from, #12254, Addgene, (Zhao et al., 2019)] expressing ACE2 (cDNA from Dr. Markku Varjosalo). Six hours after transfection, media was replaced with fresh Dulbecco’s Modified Eagle Medium (DMEM) containing high glucose (Sigma–Aldrich) supplemented with 10% fetal calf serum (FCS). Culture supernatant was collected 2 days after transfection and passed through a 0.22-micron filter. The supernatant containing TMPRSS2 lentivector was added to human embryonic kidney cells, HEK293T, seeded on 6-well plates. Following 2 days of selection with 15 μg/ ml of blasticidin (Invitrogen), the cells were incubated with the medium containing the ACE2 lentivector, and supplemented with 3 μg/ ml puromycin (Sigma–Aldrich). After selection with puromycin, single cell lines were established by serial dilution. Both the double-transduced pool and the cloned cell lines were analyzed with SDS-PAGE using an anti-V5 antibody (#MA5-15253, Invitrogen) for TMPRSS2 and an anti-ACE2-antibody (#15983, Cell Signaling Technology).

HEK293T and HEK293T-ACE2-TMPRSS2 cells were maintained in DMEM supplemented with 10% FCS, 2% L-Glutamine, and 1% penicillin/streptomycin. Human lung epithelial cell line Calu-1 was kept in Roswell Park Memorial Institute (RPMI) 1640 medium supplemented with 10% FCS, 1% L-Glutamine, and 1% penicillin/streptomycin. Another human lung epithelial cell line Calu-3 and human colorectal adenocarcinoma cell line Caco-2 were kept in Minimum Essential Medium Eagle (MEM) supplemented with 20% FCS, 1% L-Glutamine, 1% penicillin/streptomycin, and 1% MEM Non-essential Amino acid Solution (100X). VeroE6 cells (ATCC^®^ CRL-1586) were maintained in MEM supplemented with 10% FCS, 1% L-Glutamine and 1% penicillin/streptomycin. All the cell lines were incubated at 5% CO2 and 37°C.

We compared the expression level of *ACE2, TMPRSS2, FURIN* and *GAPDH* with qPCR in all the cell lines (Supplementary Figure S1) by using human specific primers (Supplementary Table S1).

### Production of Luciferase-Encoding Lentiviral Vector Pseudotyped with WT and Mutant SARS-CoV-2 S-Glycoprotein, and VSV-Glycoprotein

HEK293T cells grown in T175 flask were transfected by using TransIT-2020 reagent (Mirus Bio) with p8.9NDSB (Berthoux et al., 2004), pWPI-puro expressing *Renilla* luciferase, pEBB-GFP, and pCAGGS, an expression vector containing the SARS-CoV-2 spike glycoprotein cDNA of the Wuhan-Hu-1 reference strain (NC_045512.2). The last 18 codons of spike glycoprotein were deleted to enhance the plasma membrane transport. Pseudotyped viruses harbouring the glycoprotein of vesicular stomatitis virus (VSV-G) were produced following the same protocol by using VSV-G envelope expression vector pMD2.G (#12259, Addgene). The culture media was replaced 12–16 h after transfection with fresh DMEM High Glucose (Sigma–Aldrich) supplemented with 10% FBS. The supernatant containing the SARS-CoV-2 spike glycoprotein- or VSV-G-harboring pseudoviruses was collected 48 h after transfection, and passed through a 0.22-micron filter.

Mutations in RBD regions were generated by synthetic DNA (Integrated DNA Technologies) using PvuII and HpaI restriction sites. Lentiviral vectors pseudotyped with mutant spikes were produced by following a similar protocol as the wild type particles with minor changes. Instead of pWPI-puro expressing *Renilla* luciferase, pWPI plasmid carrying both GFP and *Renilla* luciferase were used to eliminate the use of pEBB-GFP plasmid.

### Detection of Viral Infection by Pseudotyped SARS-CoV-2 or VSV-G Viruses and Native SARS-CoV-2

**FIGURE 1:**
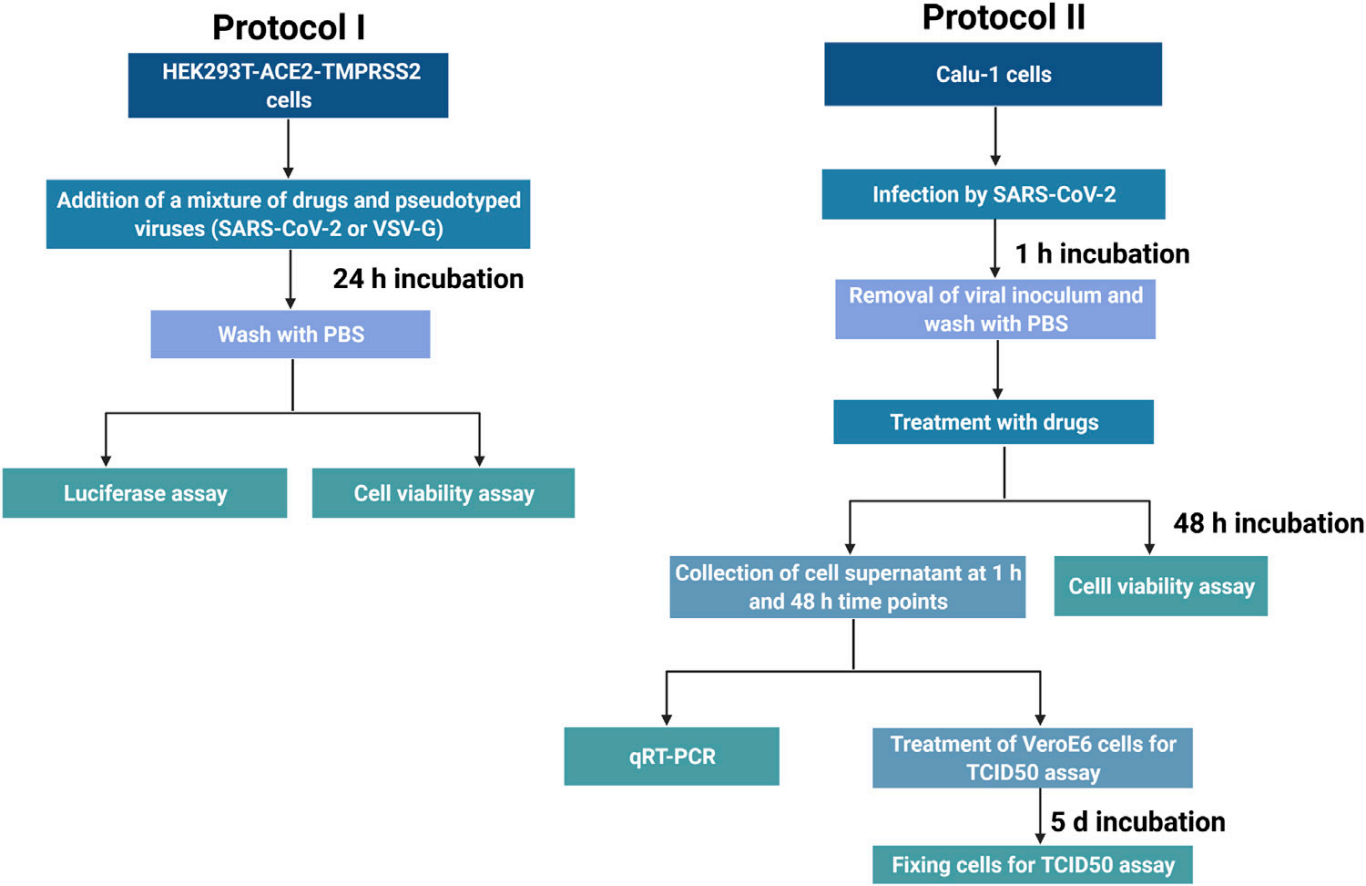
Protocols used in the study. Protocol I represents the experiments in HEK293T-ACE-TMPRSS2 cell line, while Protocol II corresponds to the experiments conducted in Calu-1 cell line. created with BioRender.com.

In the Protocol I (Figure 1), infection and treatment of HEK293T-ACE2-TMPRSS2 cells was used. Because of the replication-deficient nature of pseudo-viruses, we aimed at understanding the effect of drugs on the initial viral entry to cells. Therefore, we exposed cells to the compounds and the pseudoviruses simultaneously.

HEK293T-ACE2-TMPRSS2 cells were cultured in poly-L-lysine coated 96-well plates (ViewPlate 96, PerkinElmer Life Sciences). Next day, the cells received varying concentrations of listed drugs (Table 1) and pseudotyped lentiviruses harboring S-protein of SARS-CoV-2 or glycoprotein of VSV. Following 24 h incubation with drugs and viral particles, cells were washed once with PBS. After cells were lysed for 15 min at RT, *Renilla* luciferase reporter was used to measure viral entry. For this purpose, luciferase activity was measured with a plate reader employing dispenser feature (Varioskan Flash, ThermoFisher Scientific) after substrate addition to each well (*Renilla* luciferase Assay System, E2820, Promega or Coelenterazine native, cat#303, Nanolight Technology). In order to measure background signal, uninfected cells and empty wells were included into the assay plate.

Calu-1 cells were used in the Protocol II (Figure 1) to address the drug effect on the replication and secondary wave of infection by SARS-CoV-2 virus. Protocol II, while complementing protocol I, addresses the efficiency of drug treatment on infection by the native virus. After identifying selective serotonin reuptake inhibitors (SSRIs), selective norepinephrine reuptake inhibitor (NRI), serotonin - norepinephrine reuptake inhibitor (SNRI), tricyclics, and antipsychotics that reduced the pseudoviral infection in HEK cells, we shortlisted candidates to be tested in Calu-1 cells with the native virus. Therefore, we ensured that there is, at least, one representative from one of these groups (Table 1). All work with infectious SARS-CoV-2 virus was conducted in a Biosafety Level 3 (BSL-3) laboratory of UH at Haartman Institute. SARS-CoV-2 virus isolates (wild type, B.1.1.7, B.1.351, and B.1.617.2), obtained from nasopharyngeal swabs of patients (Cantuti-Castelvetri et al., 2020) (MOI 0.05), were incubated with Calu-1 cells in 48-well plates (40.000 cells/well) for 1 h at 37°C and 5% CO_2_, after which virus inoculum was removed and cells were washed twice with PBS. The compounds were added to the cells in six replicates, diluted in virus growth medium (VGM) (RPMI-1640 supplemented with 2% FCS, L-glutamine, penicillin and streptomycin), and 0.1% DMSO in VGM was used as control. Samples of the supernatant were collected at 1 and 48 h post infection for qRT-PCR and at 48 h for the TCID50 assay. Viral RNA was extracted using RNeasy Mini Kit (Qiagen, Germany), and SARS-CoV-2 qRT-PCR was performed using primers, probe and an *in vitro* synthesized control for RNA-dependent RNA polymerase (RdRp) as described earlier (Corman et al., 2020; Lin et al., 2021). Infectious virus titers were determined by TCID50 measurement of VeroE6 cells. Shortly, 10-fold dilutions of the samples were inoculated to VeroE6 cells, incubated for 5 days, fixed with 10% formaldehyde for 30 min RT and stained with crystal violet.

A flow chart was prepared to summarize the timeline of experiments regarding the pseudotyped viruses and the native virus (Figure 1).

### Cell Viability Assay

Cell culturing, treatment, and infection protocols were followed as described for HEK293T-ACE2-TMPRSS2 and Calu-1 cells (Figure 1). At the end of 24 h or 48 h period, cells were washed once with PBS and their viability was measured with a kit designed to quantify ATP level according to instructions of the manufacturer (CellTiterGlo 2.0 Luminescent Cell Viability Assay, Promega).

### Data and Statistical Analysis

#### Pseudotyped Viruses

The number of samples per treatment group was determined based on our earlier studies (Fred et al., 2019; Casarotto et al., 2021). Each “n” represents the number of wells used for the indicated treatment. Some drugs were tested in the same plate, therefore, the same control group values were used for the analysis. Data were normalized to control groups to avoid unwanted sources of variation, pooled together, and analyzed in GraphPad Prism 6.0 software. Pseudotyped virus experiments were analyzed by unpaired *t*-test or one-way analysis of variance (or their nonparametric equivalents) followed by Dunnett or Dunn’s post hoc tests if a significant overall effect was observed. Cell viability of non-infected and infected cells treated with indicated compounds were analyzed by two-way analysis of variance followed by Sidak’s post hoc test, again only if we observed a treatment or interaction effect between the factors. The luciferase activity readings from pseudotyped viruses (SARS-CoV-2 and VSV-G) were replotted together to run a comparative analysis. We compared the drug effect on the infection by SARS-CoV-2 spike and VSV-G pseudotyped viruses by two-way analysis of variance followed by Sidak’s multiple comparison test. The mutant pseudotyped virus experiments (infection) were also analyzed by two-way analysis of variance followed by Sidak’s multiple comparison. IC50 values were calculated in GraphPad Prism software by using the non-linear curve fitting of the data with log (inhibitor) *vs*. response-Variable slope function after conversion of drug concentrations to logarithmic scale.

Protein bands detected in Western Blotting were subtracted from background and normalized to total protein of beta actin. Data were analyzed by the two-way analysis of variance followed by Sidak’s post hoc test. Same analysis was applied to the qPCR assessment of *ACE2* and *TMPRSS2* expression in infected and non-infected HEK cells.

#### Native SARS-CoV-2 Viruses

Each “n” represents the number of wells used for the indicated treatment. The control group of some samples were the same, as they were tested in the same plate. Therefore, the same control group was represented in different plots. The plots representing changes in the genome copy number were plotted in linear scale, whereas TCID50 values were represented in log10 scale in GraphPad Prism.

For all the experiments, values of *p* < 0.05 were considered significant. Presence of any outliers was calculated by using Grubb’s test on GraphPad Prism website (https://www.graphpad.com/quickcalcs/grubbs1), and excluded from the analysis. We performed one-way ANOVA, two-way ANOVA or unpaired *t*-test, followed by Dunnet’s, Dunn’s or Sidak post hoc analysis for the experiments with the native virus. Statistical analysis were provided in Supplementary Table S2 for the corresponding figures.

## Results

### HEK293T-ACE2-TMPRSS2 Cell Line Is Responsive to Camostat Mesylate

Camostat mesylate, a clinically tested serine protease inhibitor, has been shown to inhibit the activity of TMPRSS2 and reduce the SARS-CoV-2 infection in cell lines expressing TMPRSS2 (Kawase et al., 2012; Hoffmann, et al., 2020b; Hoffmann, et al., 2020c). In order to confirm the activity of TMPRSS2 in the HEK293T-ACE2-TMPRSS2 cell line, we treated the cells with different doses of camostat mesylate for 24 h and measured the level of viral infection. The infection of HEK293T-ACE2-TMPRSS2 cell line by the pseudotyped lentiviruses was significantly reduced suggesting that these cells express TMPRSS2 and the activity of this protease is blocked by camostat mesylate (Supplementary Figure S2).

### Antidepressant Drugs Show Antiviral Activity Against Pseudotyped Viruses and the First wave (B.1) Lineage of SARS-CoV-2

**FIGURE 2:**
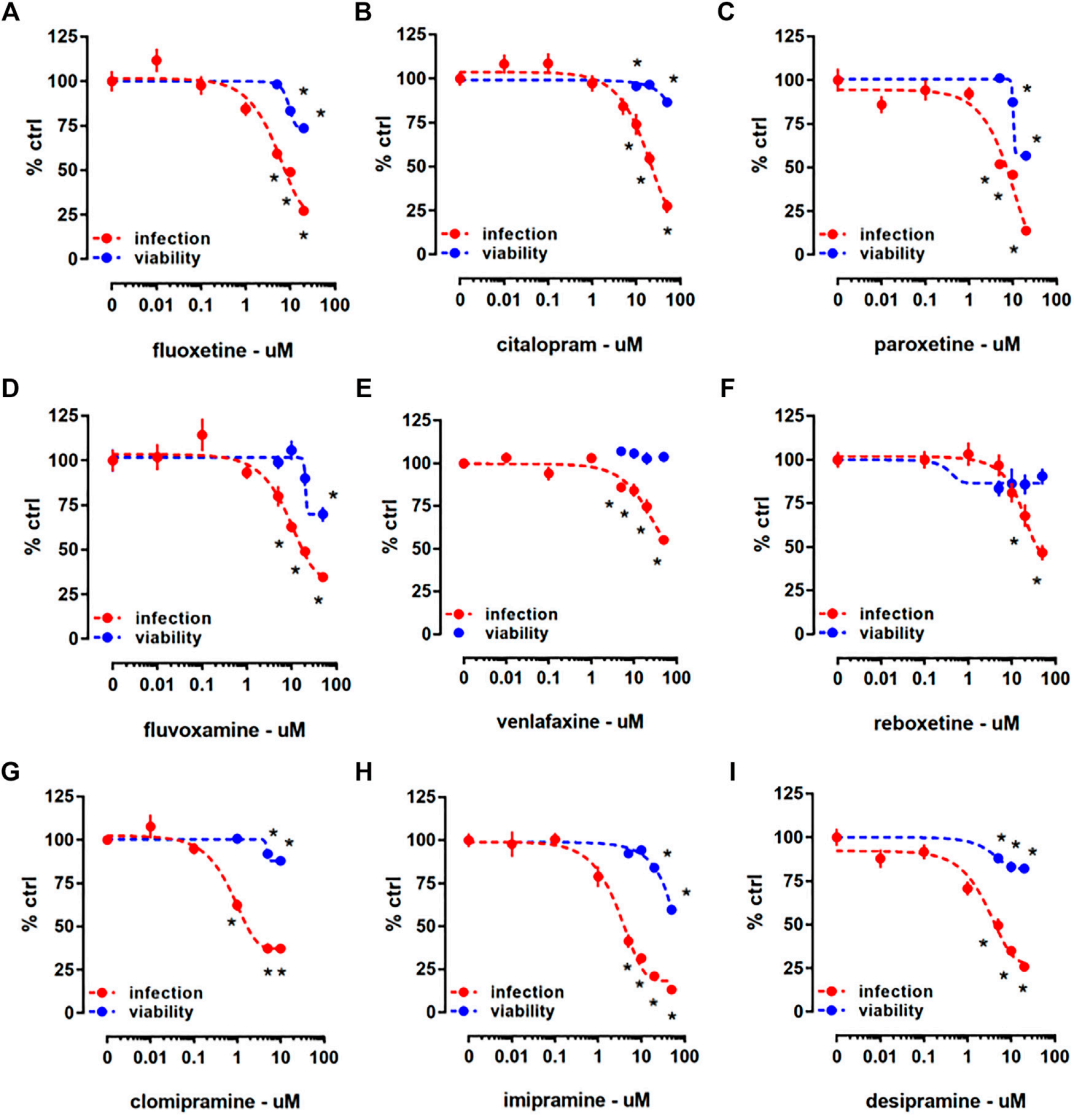
The effect of antidepressant drugs on HEK293T-ACE2-TMPRSS2 cells challenged with SARS-CoV-2 pseudotyped viruses. Treatment with **(A)** fluoxetine (luciferase assay: *n* = 18–26; cell viability: *n* = 6; IC50 = 5.992 µM), **(B)** citalopram (luciferase assay: *n* = 16–22; cell viability: *n* = 6; IC50 = 27.51 µM), **(C)** paroxetine (luciferase assay: *n* = 12; cell viability: *n* = 5; IC50 = 12.55 µM), **(D)** fluvoxamine (luciferase assay: *n* = 14–24; cell viability: *n* = 6; IC50 = 10.54 µM), **(E)** venlafaxine (luciferase assay: *n* = 11–12; cell viability: *n* = 5; IC50 = 36.35 µM), **(F)** reboxetine (luciferase assay: *n* = 12; cell viability: *n* = 6; IC50 = 17.69 µM), **(G)** clomipramine (luciferase assay: *n* = 12–24; cell viability: *n* = 6; IC50 = 0.75 µM), **(H)** imipramine (luciferase assay: *n* = 6–18; cell viability: *n* = 6; IC50 = 3 µM), and **(I)** desipramine (luciferase assay: *n* = 12; cell viability: *n* = 5; IC50 = 8.097 µM) significantly reduced luciferase reporter activity. At higher concentrations, all the compounds, except **(E)** venlafaxine and **(F)** reboxetine reduced ATP levels in the luminescent cell viability assay after 24 h incubation. *p < 0.05 from control group (0). Data represented as mean ± SEM.

In the Protocol I, HEK293T-ACE2-TMPRSS2 cells were treated with a mixture of pseudotyped viral particles and one of the following antidepressants: fluoxetine, citalopram, paroxetine, fluvoxamine, venlafaxine, reboxetine, clomipramine, imipramine, or desipramine (Table 1) for 24 h. We found that all the drugs significantly reduced the viral infection, as measured by luciferase reporter activity (Figure 2). According to the cell viability assay where ATP level was measured in infected HEK293T-ACE2-TMPRSS2 cells, all the tested compounds, except venlafaxine (Figure 2E) and reboxetine (Figure 2F), were slightly toxic at higher concentrations (Figure 2). Combination of infection with drug treatment did not induce any further toxicity (Supplementary Figure S3). We also examined the changes in mRNA level of *ACE2* and *TMPRSS2* by the viral infection combined with 24 h of fluoxetine (10 µM), clomipramine (10 µM), and chlorpromazine (5 µM) treatment (Supplementary Figure S4). The *ACE2* expression remained unaltered upon fluoxetine treatment, while clomipramine in infected cells, and chlorpromazine in non-infected cells induced a reduction of this target (Supplementary Figure S4A). *TMPRSS2* level was increased by fluoxetine in non-infected and infected cells, while the other compounds remained ineffective (Supplementary Figure S4B). Furthermore, by addressing the protein levels (Supplementary Figure S4C), we found that fluoxetine does not exert any effect on the ACE2 expression (Supplementary Figure S4D), while TMPRSS2 is significantly increased in non-infected cells after 24 h (Supplementary Figure S4E). Despite not being statistically significant, fluoxetine also increased TMPRSS2 in the infected cells (Supplementary Figure S4E).

**FIGURE 3:**
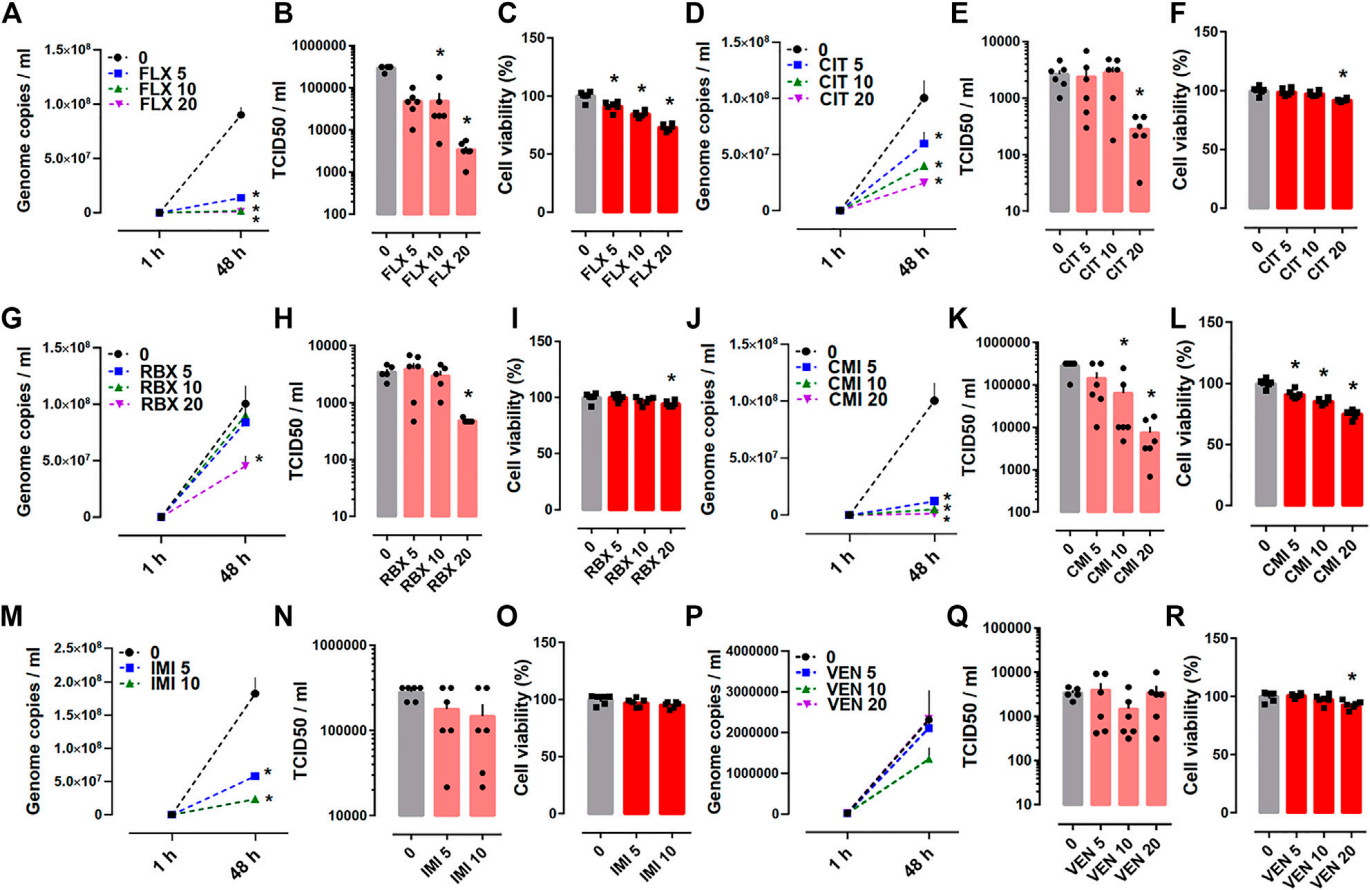
The effect of antidepressant drugs on SARS-CoV-2 (B.1) infection in Calu-1 cells. **(A,B)** Fluoxetine (*n* = 6), **(D,E)** citalopram (*n* = 4–6), **(G,H)** reboxetine (*n* = 5–6), and **(J,K)** clomipramine (*n* = 6) reduced the genome copy number of SARS-CoV-2 measured by qRT-PCR and also the amount of infectious viral particles detected by TCID50 assay after 48 h of treatment. **(M)** Imipramine (*n* = 6) reduced the genome copy number, while **(N)** failed to reduce TCID50 (*n* = 6). **(P,Q)** Venlafaxine (*n* = 5–6) was ineffective for changing the genome copy number of SARS-CoV-2 and TCID50. **(C,F,I,L,R)** All the compounds, **(O)** except imipramine, reduced the viability of Calu-1 cells infected with the native SARS-CoV-2 (*n* = 6). Genome copies were plotted in linear scale, while TCID50 values in log scale. Fluoxetine: FLX, Citalopram: CIT, Reboxetine: RBX, Clomipramine: CMI, Imipramine: IMI, Venlafaxine: VEN. *p < 0.05 from control group (0) at corresponding time point. Data represented as mean ± SEM.

In the Protocol II experiments, SARS-CoV-2 infected Calu-1 cells were treated with fluoxetine, reboxetine, clomipramine, imipramine, citalopram or venlafaxine up to 48 h at concentrations of 5, 10, and 20 µM. The base level of viral RNA that was measured 1 h after drug treatment revealed no significant changes between control and treatment groups (Figure 3). However, at 48 h after infection, fluoxetine (Figures 3A,B), citalopram (Figures 3D,E), reboxetine (Figures 3G,H), and clomipramine (Figures 3J,K) clearly reduced the genome copies of SARS-CoV-2 and infectious virus particles measured by TCID50 assay. Despite reducing the genome copies (Figure 3M), imipramine failed to effect the TCID50 (Figure 3N).Venlafaxine was ineffective on reducing the viral replication and infectious particles of native SARS-CoV-2 (Figures 3P,Q). Viability of Calu-1 cells was measured in infected cells after 48 h treatment with the compounds. All compounds, except for imipramine, decreased the cell viability (Figures 3C,F,I,L,O,R).

### Antipsychotics can Decrease the Viral Infection

**FIGURE 4:**
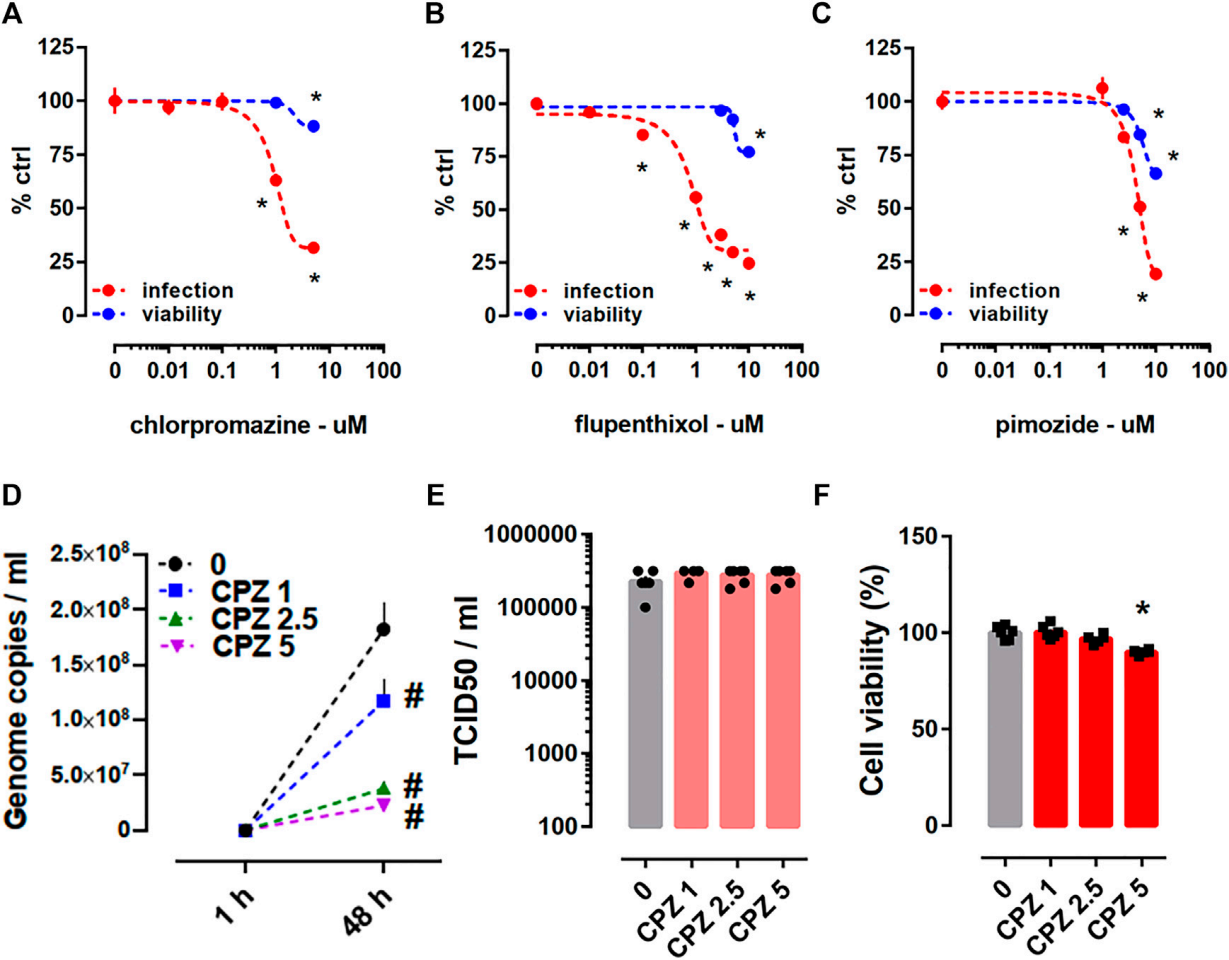
Antiviral activity of antipsychotics in HEK293T-ACE2-TMPRSS2 cells challenged with pseudotyped viruses and in Calu-1 cells infected with SARS-CoV-2 (B.1). Decline of luciferase reporter activity was observed at multiple doses of **(A)** chlorpromazine (luciferase assay: *n* = 8–20; cell viability: *n* = 8; IC50 = 0.972 µM), **(B)** flupenthixol (luciferase assay: *n* = 12; cell viability: *n* = 5; IC50 = 1.072 µM), and **(C)** pimozide (luciferase assay: *n* = 12; cell viability: n = 5; IC50 = 4.539 µM) following 24 h incubation suggesting reduced infection. Treatment of cells with **(A)** chlorpromazine, **(B)** flupenthixol, and **(C)** pimozide combined with viral infection decreased cell viability indicated by the ATP level. **(D)** Treatment with chlorpromazine for 48 h decreased the genome copy number of SARS-CoV-2 (*n* = 5–6) in qRT-PCR, but **(E)** failed to reduce the amount of infectious virus in TCID50 assay (*n* = 6). **(F)** The highest dose of chlorpromazine treatment increased the toxicity in infected Calu-1 cells indicated by the ATP level. Genome copies were plotted in linear scale, while TCID50 values in log scale. Chlorpromazine: CPZ. *p < 0.05 from control group (0). #*p* < 0.05 from control group (0) at corresponding time point. Data represented as mean ± SEM.

The antiviral activity of antipsychotics chlorpromazine, flupenthixol, and pimozide were tested through the Protocol I in HEK293T-ACE2-TMPRSS2 cells against the pseudotyped viruses harboring SARS-CoV-2 spike protein (Table 1). Following 24 h incubation, all of these compounds were able to prevent viral infection, although we also observed slight reduction of cell viability (Figures 4A–C).

Chlorpromazine (1, 2.5, and 5 µM) was also tested through the Protocol II in SARS-CoV-2 (B.1 lineage) infected Calu-1 cells. Treatment for 48 h with all the tested concentrations of chlorpromazine were able to reduce the total amount of virus (Figure 4D), but we failed to observe diminished amount of infectious virus after 48 h treatment (Figure 4E). There was a slight but significant reduction in the viability of infected Calu-1 cells after 48 h treatment with chlorpromazine (5 µM) (Figure 4F).

### Some Psychoactive Drugs Fail to Prevent the Infection by the Pseudotyped Viruses

Ketamine, used for many decades as anesthetic drug in clinics, has been shown to induce antidepressant-like effects when administered in sub-anesthetic doses (Berman et al., 2000; Zarate et al., 2006; Abdallah et al., 2015). Therefore, we next tested whether ketamine and ketamine metabolites (2S,6S-HNK and 2R,6R-HNK), which have also received attention as rapid-acting antidepressants during recent years (Abdallah et al., 2015; Zanos et al., 2018), might also inhibit infection by the pseudotyped virus. We found that neither ketamine nor the metabolites were effective in reducing the viral infection within 24 h of incubation period (Supplementary Figures S5A–C). Furthermore, other classical antidepressant drugs, phosphodiesterase-4 inhibitor rolipram and monoamine oxidase inhibitor phenelzine (Table 1) also failed to prevent the viral entry (Supplementary Figures S5D,E). We also tested carbamazepine, an anticonvulsant, which is also used as mood stabilizer for the treatment of bipolar disorder (Mitchell & Malhi, 2002). This compound also failed to change the viral infection in ACE2/TMPRSS2-expressing HEK cells after 24 h of treatment (Supplementary Figure S5F).

### Some of the Tested Drugs Show SARS-CoV-2 Spike Specificity

We used pseudotyped viruses harboring glycoprotein of vesicular stomatitis virus (VSV) to address the specificity and potential viral-entry independent effects of tested compounds. HEK293T-ACE2-TMPRSS2 cells were treated with a mixture of VSV-G pseudotyped viruses and drugs including fluoxetine, citalopram, paroxetine, fluvoxamine, reboxetine, clomipramine, desipramine, chlorpromazine, flupenthixol, or pimozide for 24 h. Viral infection was quantified by measuring the luciferase activity (Supplementary Figure S6; Supplementary Figure S7). While fluoxetine (Supplementary Figure S6A), clomipramine (Supplementary Figure S6C), chlorpromazine (Supplementary Figure S6E), flupenthixol (Supplementary Figure S6G), pimozide (Supplementary Figure S6I), and reboxetine (Supplementary Figure S6K) reduced the infection by the VSV-G pseudotyped viruses; citalopram (Supplementary Figure S7A), desipramine (Supplementary Figure S7C), paroxetine (Supplementary Figure S7E), and fluvoxamine (Supplementary Figure S7G) remained ineffective. Slight reduction of cell viability was observed in infected HEK293T-ACE2-TMPRSS2 cells after fluoxetine, clomipramine, chlorpromazine, pimozide, and reboxetine treatment (Supplementary Figure S6), while flupenthixol, citalopram, desipramine, paroxetine, or fluvoxamine did not exert a toxic effect (Supplementary Figure S6; Supplementary Figure S7).

We compared the effectiveness of the tested antidepressants and antipsychotics against SARS-CoV-2 spike- and VSV-G-pseudotyped viruses. We compiled the data from these viruses, and found higher effectiveness of drugs in reducing the luciferase activity in SARS-CoV-2 pseudoviruses compared to VSV-G (Supplementary Figures S6B,D,F,H,J,L; Supplementary Figures S7B,D,F,H).

### Fluoxetine Remains Effective Against Pseudotyped Viruses Harboring the S Protein RBD Mutations and Native Variants B.1.1.7 (Alpha), B.1.351 (Beta), and B.1.617.2 (Delta)

**FIGURE 5:**
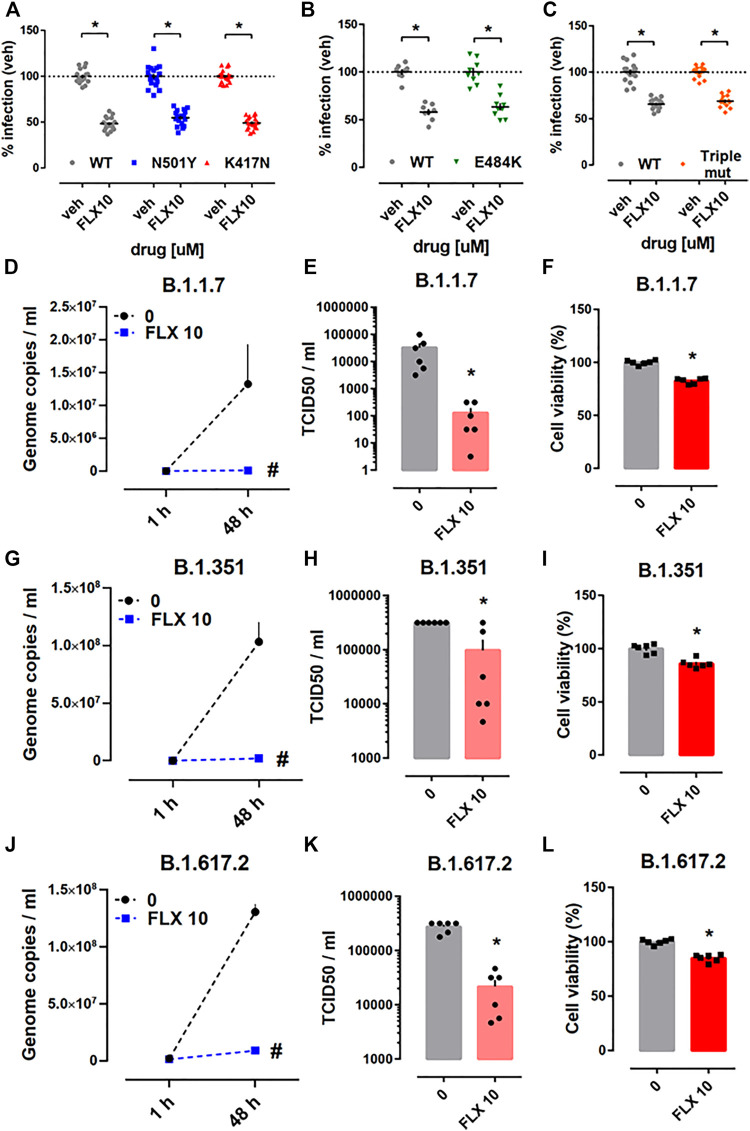
Antiviral activity of fluoxetine against the mutant pseudo-viruses and the native variants of SARS-CoV-2. Treatment with fluoxetine (10 μM, 24 h) significantly reduced the luciferase reporter activity in HEK293T-ACE2-TMPRSS2 cells infected with **(A)** WT (grey, *n* = 16), N501Y (blue, *n* = 18), K417N (red, *n* = 18), **(B)** E484K (green, *n* = 8–9) and **(C)** triple mutant (N501Y/K417N/E484K, orange, *n* = 13–16), viral particles. Fluoxetine (10 µM) reduced genome copy number and TCID50 in Calu-1 cells infected with **(D,E)** B.1.1.7 (alpha), **(G,H)** B.1.351 (beta), and **(J,K)** B.1.617.2 (delta) variants, measured by qRT-PCR and TCID50 assay, respectively (*n* = 6). **(F,I,L)** Fluoxetine also slightly reduced the Calu-1 viability in cells infected with the variants (*n* = 6) measured by the ATP levels. Fluoxetine: FLX and wild type: WT. *p < 0.05 from control group (veh). #*p* < 0.05 from control group (0). Data represented as mean ± SEM.

Next, we addressed the effectiveness of fluoxetine against the pseudotyped viruses carrying mutations in the S protein that have been observed in some of the emerging SARS-CoV-2 variants. We found that fluoxetine (10 µM) treatment for 24 h was effective against pseudotyped viruses carrying single point mutations in their S protein (N501Y, K417N, or E484K) (Figures 5A,B), and also a triple mutant harboring combination of these three point mutations (N501Y/K417N/E484K) (Figure 5C). Calu-1 cells which were infected with B.1.1.7 (alpha), B.1.351 (beta), or B.1.617.2 (delta) variants, and challenged with fluoxetine (10 µM) showed reduced amount of genome copies (Figures 5D,G,J) and infectious virus (Figures 5E,H,K). The viability of Calu-1 cells infected with these variants and treated with fluoxetine for 48 h was slightly reduced (Figures 5F,I,L).

## Discussion

In the present study, we identified the potential of some widely consumed antidepressant and antipsychotic drugs against the infection by SARS-CoV-2 (B.1) and the variants B.1.1.7 (alpha), B.1.351 (beta), and B.1.617.2 (delta). The experiments using pseudotyped viruses to infect HEK293T cells overexpressing ACE2 and TMPRSS2 were aimed at identifying the direct effect of drugs on the entry phase of the viral infection, as we added the virus and drugs around the same time. The outcome measure by luciferase assay was a direct indication of the number of infected cells, due to replication-deficient nature of pseudotyped viruses. In experiments with human respiratory/lung epithelial cells and native virus, Calu-1 cells were first exposed to the SARS-CoV-2 virus, which was followed by the removal of this inoculum and addition of drugs to be tested. In this scenario, drugs do not necessarily interfere with the initial infection of cells by the virus, which was at low multiplicity of infection (MOI 0.05), but with viral particle production and infection of the neighboring cells. The decrease in viral replication that we observed after treating the cells for 48 h with the tested drugs and the reduction of TCID50 as a secondary outcome could be caused by 1) the blockade of virus entry during the subsequent waves of Calu-1 cell infection; 2) a direct effect on the mechanisms of viral replication, packaging and release of new viral particles; 3) an indirect effect through the regulation of innate immunity; or 4) a combination of the above effects. The first alternative, that the drugs interfere with viral infection of Calu-1 cells later on when the cells release newly packed viruses to medium thereby explaining the decline of viral genome copies and TCID50, would also be compatible with our findings with the pseudotyped virus.

By testing pseudotyped viruses harboring the VSV-G, we found that some compounds, including fluoxetine, clomipramine, chlorpromazine, flupenthixol, pimozide, and reboxetine, counteract the infection in HEK-ACE2-TMPRSS2 cells suggesting that these drugs are not specific for SARS-CoV-2 spike protein. On the other hand, the effect was significantly smaller against the VSV-G pseudoviruses compared to the SARS-CoV-2 pseudoviruses. Although the reduction of viability may have contributed to the diminished VSV-G infection, it should be noted that these compounds are effective against the entry of other viruses, as this has been reported in studies testing clomipramine and flupenthixol against Ebola virus (Johansen et al., 2015). Moreover, as these drugs also counteract VSV-G pseudoviruses, it is unlikely that mechanism of action is on the luciferase transcription. Interestingly, fluvoxamine, paroxetine, desipramine, nor citalopram exerted any effect on VSV-G infection suggesting the SARS-CoV-2 spike specificity of these compounds.

Accumulating data suggest that the psychoactive drugs that have been categorized as functional inhibitors of acid sphingomyelinase activity (FIASMA) (Kornhuber et al., 2010; Kornhuber et al., 2011) can significantly reduce the SARS-CoV-2 infection through cellular events associated with cholesterol-trapping, luminal pH changes in endolysosomal compartments, and ceramide manipulation (Carpinteiro et al., 2020; Schloer et al., 2020). FIASMA compounds, other than psychoactive drugs, have been reported to reduce SARS-CoV-2 infection *in vitro* (i.e., ambroxol, a mucolytic drug) (Carpinteiro et al., 2021), and also diminished the risk of intubation or death from COVID-19 in patients (i.e., cardiovascular system medications) (Hoertel, et al., 2021a). Most of the compounds we tested in the present study that showed antiviral activity are considered FIASMAs. On the other hand, we found that non-FIASMA compounds reboxetine, in both HEK and Calu-1 cells, and venlafaxine (Kornhuber et al., 2011; Zeitler et al., 2019), in HEK cells, demonstrate antiviral activity. However, the failure of venlafaxine against the native SARS-CoV-2 was unexpected. The data prompted us to assume that HEKs and Calu-1 cells might show differential expression and/or recruitment of distinct molecular targets for the infection by pseudoviruses and the native SARS-CoV-2, respectively. Therefore, availability or absence of specific targets might affect the drug response. Altogether, inhibition of ASM by these drugs can only partially explain the role of psychoactive drugs for the reduction of SARS-CoV-2 infection. Recently, some of these FIASMA compounds, including cationic amphiphilic drugs (CADs), have been suggested to induce phospholipidosis, which can explain the antiviral action of these drugs (Tummino et al., 2021).

Another mechanism of action proposed for these compounds, is the Sigma-1 receptor (S1R) agonism, as the activation of this receptor is known to regulate cytokine production (Lenze et al., 2020). Many psychoactive drugs bind to S1R, including fluvoxamine, sertraline, fluoxetine, citalopram, imipramine, paroxetine, and desipramine (Narita et al., 1996; Sukhatme et al., 2021). However, venlafaxine, one of the compounds that showed antiviral activity in our study in HEK cells, has a very weak affinity to this chaperone (Ishima et al., 2014). Moreover, ketamine can bind to both S1R and S2R (Robson et al., 2012), but this drug was ineffective as an antiviral agent in the present study. Therefore, S1R agonism and the regulation of cytokine levels through this chaperon does not appear to fully explain how these drugs act as antiviral agents.

Our data on chlorpromazine are in agreement with the earlier studies that have demonstrated antiviral activity against other coronaviruses (Cong et al., 2018; Dyall et al., 2014; Wilde et al., 2014). Other antipsychotics, flupenthixol and pimozide, identified as inhibitors of pseudotyped viral infection of HEK cells in our study, have also been confirmed by others as antiviral agents (Drayman et al., 2020; Yang et al., 2020). Pimozide, tested by computational docking analysis and *in vitro* assays, has been suggested to inhibit main protease of SARS-CoV-2 (M^Pro^) (Vatansever et al., 2020).

Emergence of the novel variants raises concern on the effectiveness of currently available SARS-CoV-2 vaccines for the neutralization of these variants (Callaway, 2021; Callaway and Ledford, 2021; Fontanet et al., 2021). Recent *in vitro* studies reported that some of these vaccines remain effective against the variants carrying a set of mutations (Muik et al., 2021; Wu et al., 2021; Xie et al., 2021); however, others have shown decreased effectiveness, particularly to those carrying the E484K mutation (Wadman, 2020; Callaway and Mallapaty, 2021; Collier et al., 2021; Wang et al., 2021). Our data suggest that fluoxetine remains effective against the pseudotyped viruses harboring single mutations (N501Y, K417N, E484K), or a triple mutation (N501Y/K417N/E484K) present in beta and gamma variants. In line with this, inhibitory effect of fluoxetine persists against the SARS-CoV-2 variants alpha, beta, and delta. Therefore, it is plausible to argue that antidepressants, as well as related-psychoactive compounds can be considered as an alternative treatment method for people infected with SARS-CoV-2.

One of the limitations of our study is that inferring the drug concentrations required for the inhibition of SARS-CoV-2 infection at the clinical level from *in vitro* data is difficult, as these drugs accumulate in different organs, including brain and lungs, and show variance among individuals (Karson et al., 1993; Bolo, 2000; Johnson et al., 2007). Samples collected from pilot fatalities show 19.6 μg/ g mean concentration of fluoxetine in lungs (∼7.6 µM) which is in the concentration range that is effective against SARS-CoV-2. Although further experiments are needed to address the tissue concentrations for the other molecules tested, these data suggest that due to accumulation into lung tissue (R. D. Johnson et al., 2007) (60-fold higher concentrations were found in lungs than in plasma), the concentrations tested here have been estimated to be clinically relevant against the SARS-CoV-2 infection (Eugene, 2021). We have also listed the IC50, CC50, and selectivity index (SI) parameters in Supplementary Table S3, which were calculated from the HEK cell response. We almost never reached to 50% drop in the cell viability test with the concentrations tested, which might have affected the CC50 values calculated by curve fitting. Therefore, interpretation of CC50 and SI values to suggest a safe antiviral window might be misleading.

Another interesting result we observed was the increase of TMPRSS2 in HEK cells after 24 h fluoxetine treatment. The TMPRSS2 increase by fluoxetine was detected in both non-infected and infected cells suggesting that the infection alone has no impact on this event. In fact, we expected to observe a reduction of TMPRSS2 by fluoxetine. However, the increase of TMPRSS2 after 24 h fluoxetine treatment might not be related with the antiviral action of fluoxetine. To gain an insight into this issue, testing fluoxetine with different treatment regimens is needed.

Dissecting the viral entry routes provides the opportunity for alternative strategies for prevention and management of the viral infection. Since the emergence of COVID-19, substantial progress has been made towards understanding the life cycle of SARS-CoV-2 and particularly how the virus can enter target cells (T. Tang et al., 2020). Earlier studies on viruses including SARS-CoV, Ebola, and influenza shed a light on the mechanism of viral entry and accentuated the importance of endolysosomal compartments and cholesterol (Mingo et al., 2015; Kühnl et al., 2018), which led to the recent discoveries of repurposed drugs against SARS-CoV-2 (Carpinteiro et al., 2020; Schloer et al., 2020). Alternative strategies, including the combination of compounds that can target machineries in host cells and the virus itself have also been developed to increase the effectiveness of the treatment (Schloer et al., 2021). For instance, combination of fluoxetine with remdesivir, a nucleotide analog interfering RNA-dependent RNA polymerase, was shown to be more effective than either of these compounds alone (Schloer et al., 2021).

Together with other recent studies, our data suggest that antidepressant drugs might become an additional tool against the COVID-19 pandemic, therefore, further clinical studies implicating bigger sample size and longer follow-up periods should be conducted.

## Data Availability

The original contributions presented in the study are included in the article/Supplementary Material, further inquiries can be directed to the corresponding author. Data produced in the present study is openly available in FigShare (DOI: 10.6084/m9.figshare.14248856).
