# Supplementary material for: Antidepressant and Antipsychotic Drugs Reduce Viral Infection by SARS-CoV-2 and Fluoxetine Shows Antiviral Activity Against the Novel Variants in vitro
Source: Front Pharmacol. 2022 Jan 19;12:755600. doi: 10.3389/fphar.2021.755600 (PMC8809408; doi:10.3389/fphar.2021.755600)
Supplement: Supplementary file 1 [file DataSheet1.pdf]

Supplementary Material

1 Supplementary Materials and Methods

mRNA isolation, cDNA synthesis and quantitative PCR (qPCR)

Cells were washed with PBS (1X) once and collected after incubation in Qiazol lysis reagent (QIAGEN). Lysates were incubated in chloroform at RT for 3 min and vortexed rigorously for 15 seconds. After centrifugation at 12000 rpm for 10 min, aqueous phase was transferred to clean tubes and mixed with isopropanol. The samples were centrifuged for 10 min and the supernatant was discarded. Extensive washes in 70% ethanol was followed by a final wash in 96% ethanol. Pellets were air-dried and dissolved in RNAse-free MQ. The quantity and purity of RNA samples were measured with Nanodrop (Thermo Scientific).

Maxima First Strand cDNA Synthesis Kit for RT-qPCR with dsDNAse (#K1672, Thermo Scientific) was used to synthesize cDNA according to the instructions of the manufacturer. Genomic DNA contamination was assessed by including an "RT-" control and the reagent contamination was addressed by using a "no template" control.

Human-specific primers for qPCR were retrieved from the literature (Table S1). Maxima®SYBR Green qPCR Master Mix (2X) (Thermo Scientific, #K0253) was mixed with cDNA samples according to the instructions of the manufacturer and loaded to Hard-Shell® 96-well PCR plate (BioRad) as duplicates. Thermal cycler (BioRad CFX96 Real-Time System) was used for the assay with 44 cycles of amplification. Amplicon size and quality were verified by using agarose gel electrophoresis (data not shown).

In a separate experiment, infected HEK293T-ACE2-TMPRSS2 cells were treated with fluoxetine (10 µM), clomipramine (10 µM) or chlorpromazine (5 µM) for 24 h. Following the same protocol above, qPCR was performed to measure *ACE2*, *TMPRSS2*, and *GAPDH*.

SDS-PAGE and Western Blotting

HEK293T-ACE2-TMPRSS2 cells were cultured in 24-well plates and received 10 µM fluoxetine together with SARS-CoV-2 pseudoviruses following the Protocol I. After 24 h infection, cells were lysed in NP lysis buffer [20 mM Tris-HCl, 137 mM NaCl, 10% glycerol, 50 mM NaF, 1% Nonidet P-40, 0.05 mM Na3VO4, containing a mixture of protease and phosphatase inhibitors (#P2714 and #P0044, respectively, Sigma–Aldrich)]. Following the centrifugation at 16000 xg (4°C, 15 min), supernatants were mixed with Laemmli buffer and boiled (95ºC, 5 min). Proteins were resolved in 4-12% Bis-Tris gel (NuPAGE, Thermo Fisher Scientific) and transferred to polyvinylidene difluoride membranes (*Amersham* Hybond, GE Healthcare). After a blocking step in 3% bovine serum albumin (BSA) in TBST (20 mM Tris-HCl, 150 mM NaCl, 0.1% Tween 20, pH 7.6) for 2 h at RT, the membranes were incubated overnight at 4 °C with antibodies against ACE2 (rabbit, 1:2000, #4355, Cell Signaling, AB_2797606), V5 (mouse, 1:1000, #R960-25, Invitrogen, AB_2556564), and beta actin (mouse, 1:5000, #A1978, Sigma-Aldrich, AB_476692).

HRP-conjugated secondary antibodies, goat anti-rabbit (1:5000, #170-5046, Bio-Rad) and goat anti-mouse (1:5000, #1706516, Bio-Rad) were diluted in 3% BSA in TBST. Membranes were incubated with the secondary antibodies (1h, RT) and washed several times in TBST and TBS. The chemiluminescence signal was developed with ECL and detected by a CCD camera. Quantification of bands was performed with ImageJ, FIJI.

2 Supplementary Figures and Tables

2.1 Supplementary Figures

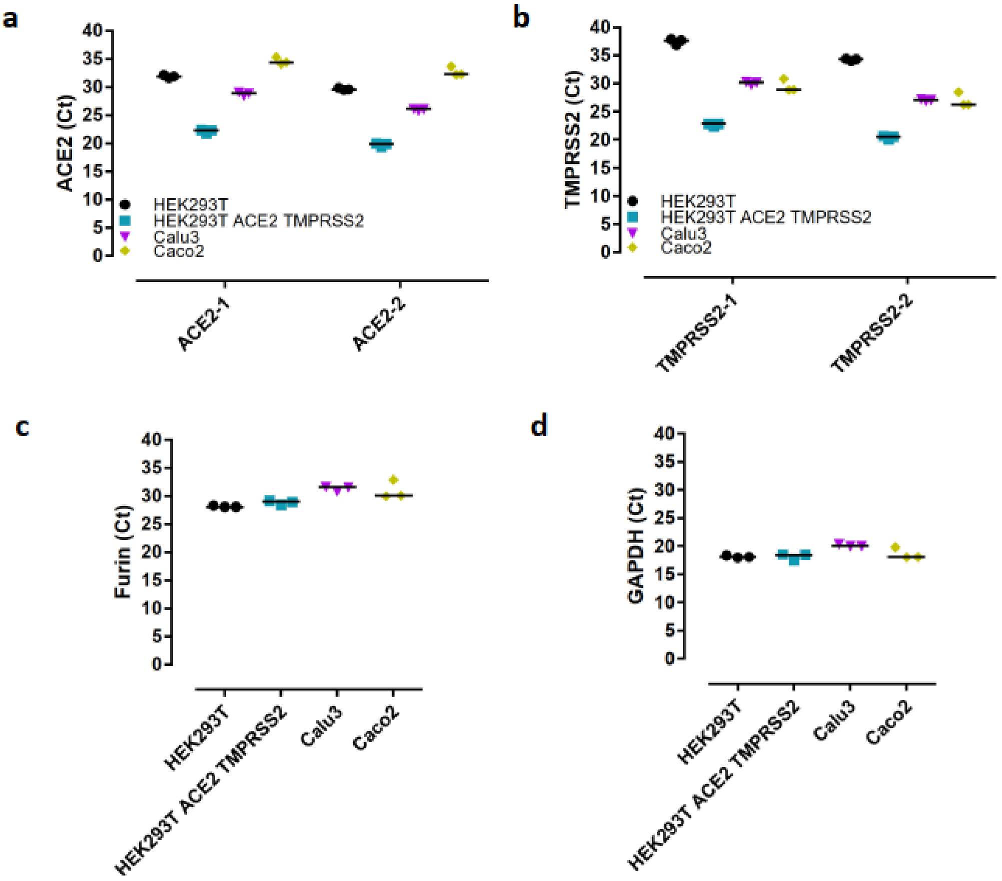

Supplementary Figure 1. qPCR validation of *ACE2, TMPRSS2, FURIN* and the housekeeping gene *GAPDH* expression in cell lines. cDNA samples collected from HEK293T, HEK293T-ACE2-TMPRSS2, Calu-3, and Caco-2 cell lines were subjected to qPCR in order to address the expression of genes which are essential for the binding, processing, and entry of SARS-CoV-2. Ct values were represented for **(a)** *ACE2* by using two sets of primers, **(b)** *TMPRSS2* by using two sets of primers, **(c)** *FURIN*, and **(d)** *GAPDH*. n= 3 for all the groups.

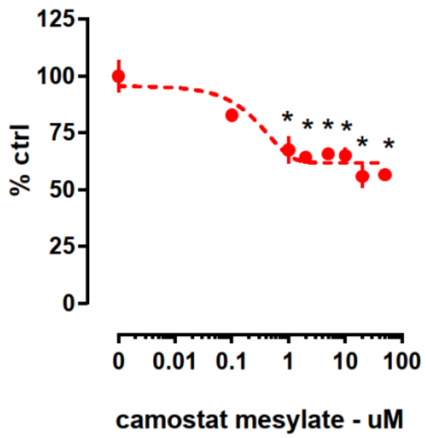

Supplementary Figure 2. Luciferase reporter activity in HEK293T-ACE2-TMPRSS2 cells challenged with pseudotyped viruses harboring SARS-CoV-2 spike protein and camostat mesylate for 24 h. Camostat mesylate induces a reduction of luciferase activity in HEK 293T-ACE2-TMPRSS2 cells (n= 6-7). *$p < 0.05$ for comparison with the control group (0). Data represented as mean ± SEM.

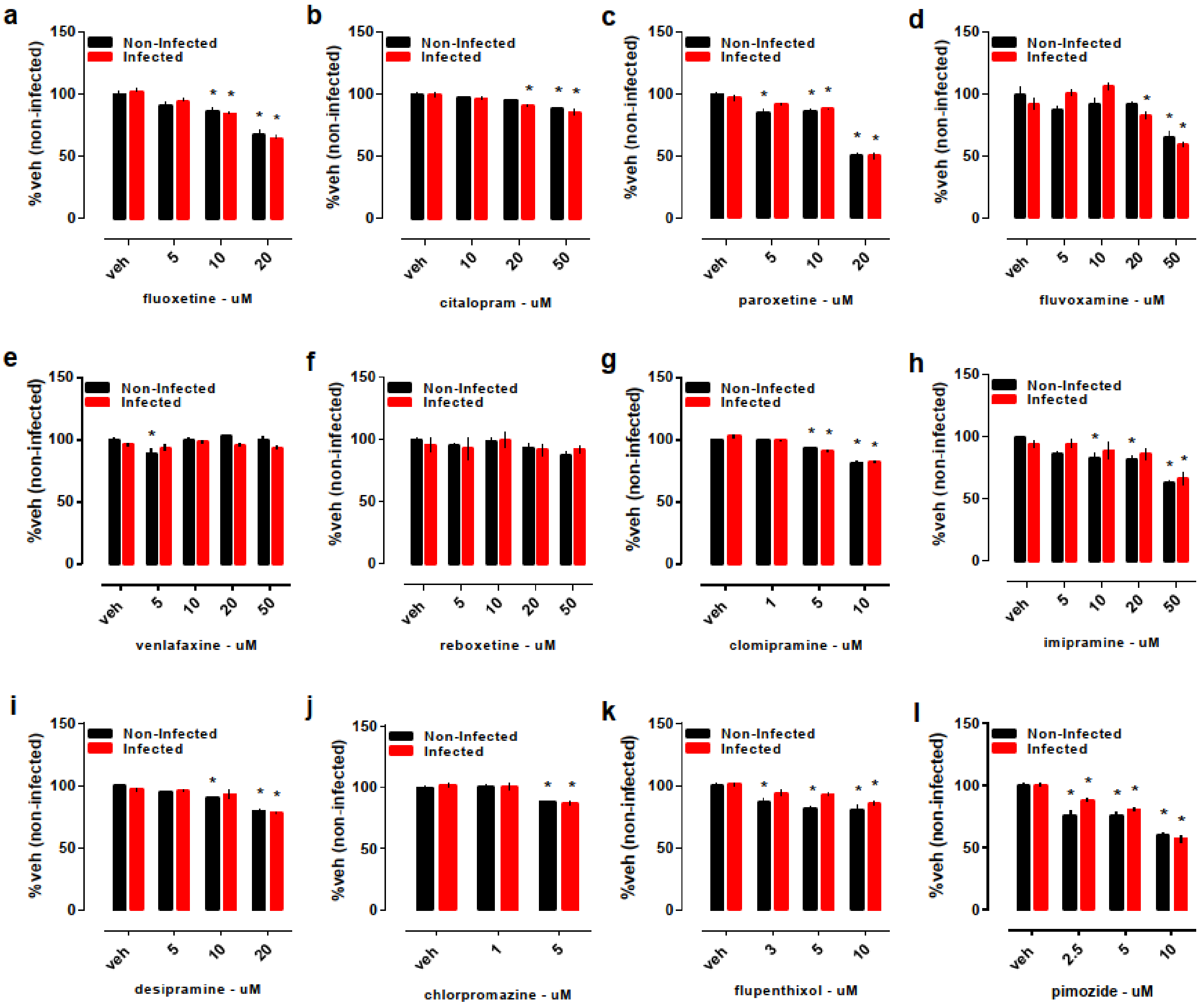

Supplementary Figure 3. Assessment of ATP level (viability) in non-infected and SARS-CoV-2 pseudovirus infected HEK-ACE2-TMPRSS2 cells combined with 24 h drug treatment. Reduction of cell viability was observed in both non-infected and infected cells after treatment with **(a)** fluoxetine, **(b)** citalopram, **(c)** paroxetine, **(d)** fluvoxamine, **(g)** clomipramine, **(h)** imipramine, **(i)** desipramine, **(j)** chlorpromazine, **(k)** flupenthixol, and **(l)** pimozide. **(f)** Reboxetine did not affect the viability of non-infected and infected cells. The infection exerted a protective effect in cells treated with **(e)** venlafaxine and **(l)** pimozide. There was also an interaction between infection and treatment in cells treated with **(d)** fluvoxamine and **(k)** flupenthixol. n=6 for all the groups. *$p < 0.05$ from control group (veh/non-infected). Data represented as mean ± SEM.

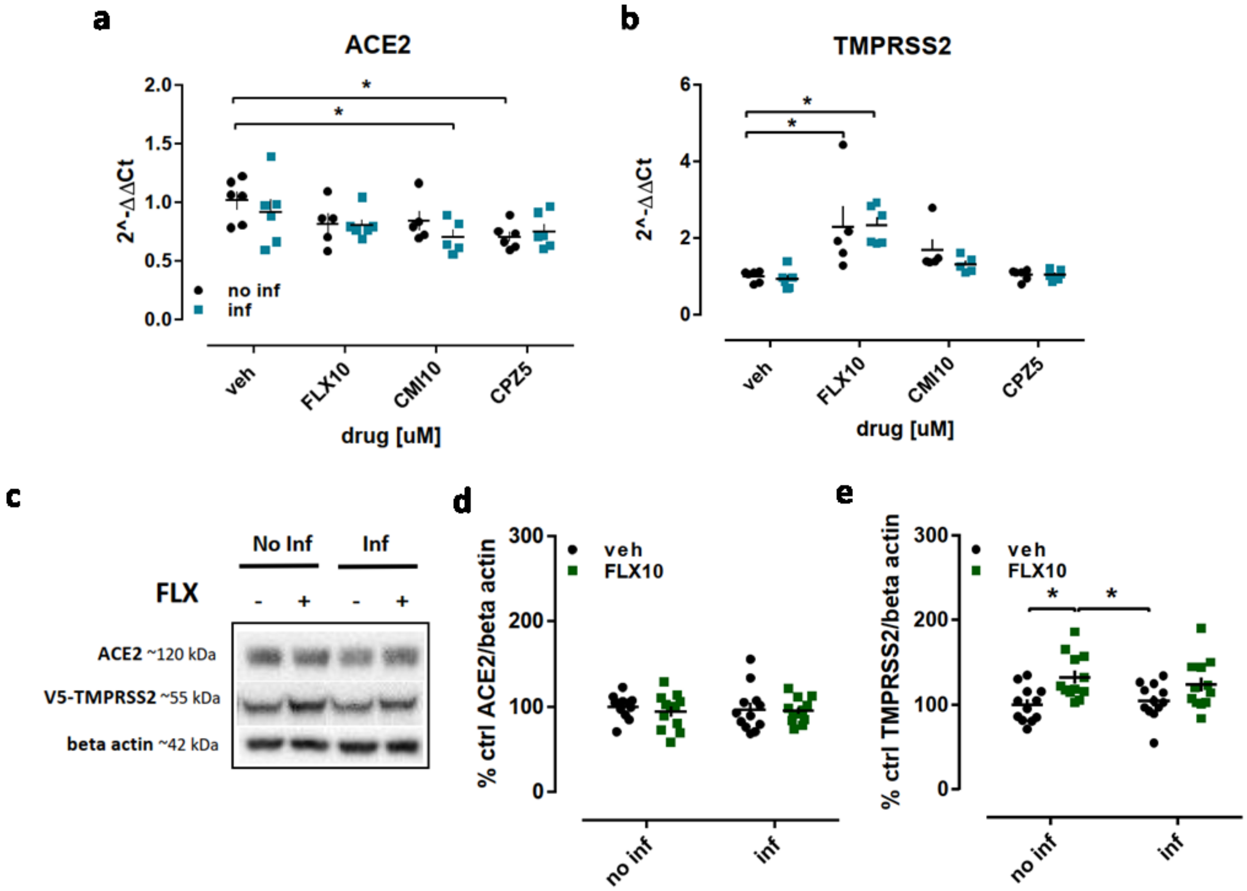

Supplementary Figure 4. qPCR and Western Blotting measurement of ACE2 and TMPRSS2 in non-infected and infected HEK293T-ACE2-TMPRSS2 cells with or without drug treatment. HEK293T-ACE2-TMPRSS2 cells were infected with pseudotyped viruses carrying the spike protein (inf) or left uninfected as control (no inf). Cells were treated with DMSO (veh), fluoxetine (10 µM), clomipramine (10 µM) or chlorpromazine (5 µM) for 24 h. **(a)** *ACE2* mRNA level was reduced following clomipramine and chlorpromazine treatments in infected and uninfected cells, respectively, while fluoxetine was ineffective (n= 5-6). **(b)** *TMPRSS2* mRNA level was increased by fluoxetine in infected and uninfected cells, while other compounds showed no effect (n=5-6). **(c)** Lysates of cells treated with fluoxetine were analyzed with Western Blotting. **(d)** Neither infection nor fluoxetine changed the level of ACE2 (n=12), while **(e)** fluoxetine increased the TMPRSS2 level in non-infected cells (n=12). *$p < 0.05$ in comparison to veh (no inf) group. Data represented as mean ± SEM.

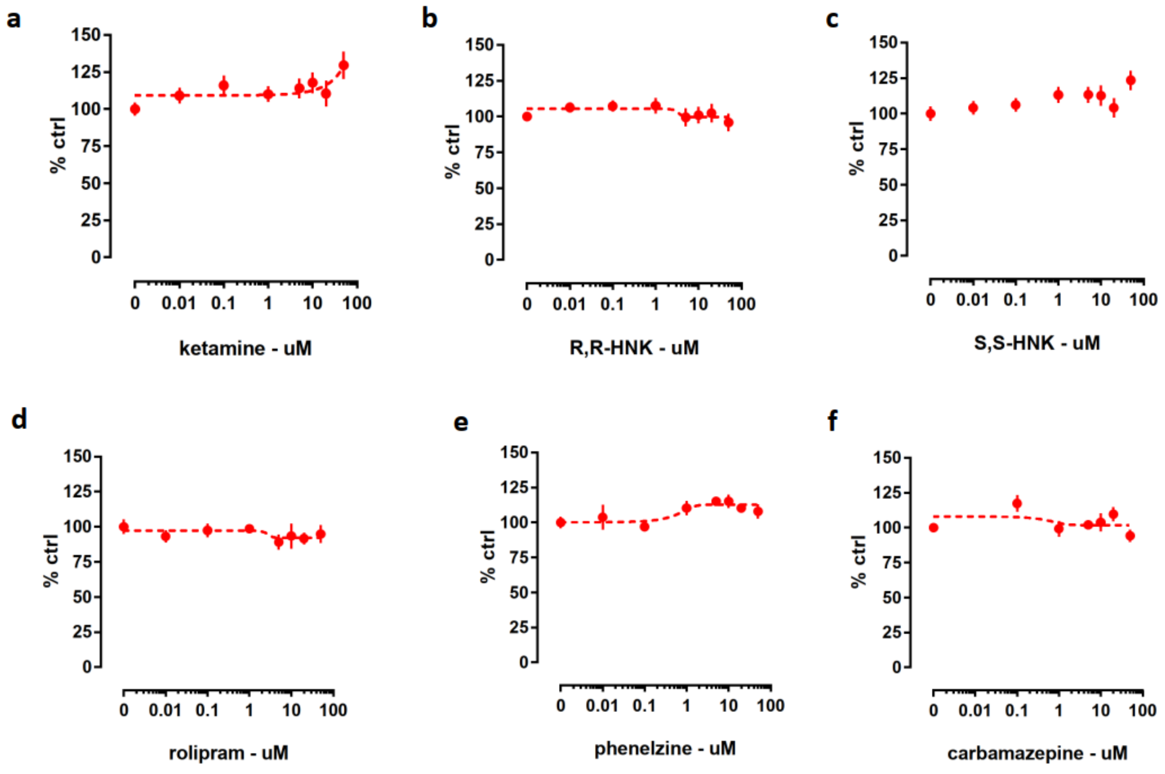

Supplementary Figure 5. Luciferase reporter activity in HEK293T-ACE2-TMPRSS2 cells 24 h after infection by SARS-CoV-2 pseudotyped viruses and treatment with indicated compounds. **(a)** Ketamine (n= 15-27) and ketamine metabolites **(b)** 2R,6R-HNK (n= 12-16) and **(c)** 2S,6S-HNK (n= 11-16) did not change the luciferase reporter activity. Other classical antidepressant drugs **(d)** rolipram (n= 6-8) and **(e)** phenelzine (n= 5-8) were also found to be ineffective. **(f)** Anticonvulsant carbamazepine (n= 6) did not change the amount of luciferase activity. Data represented as mean ± SEM.

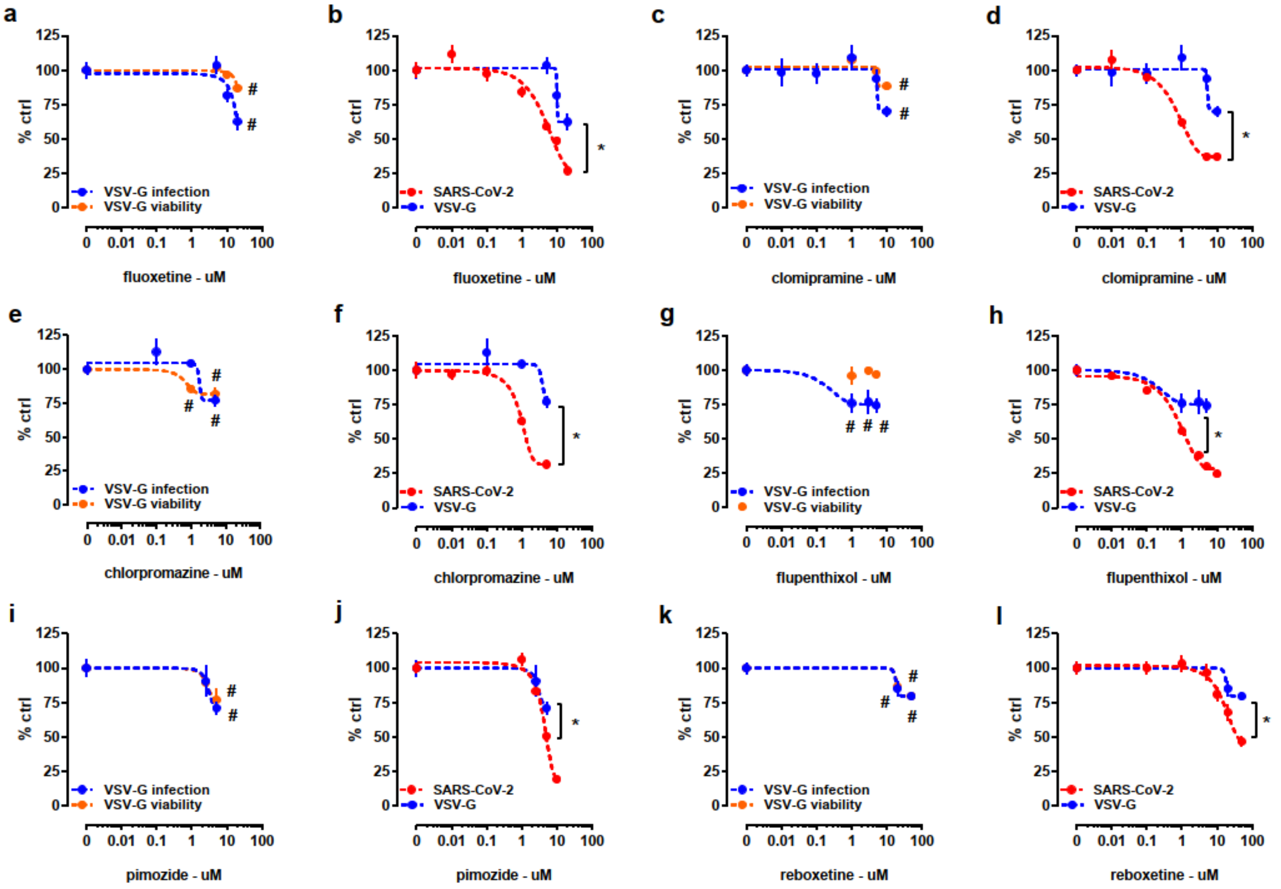

Supplementary Figure 6. Luciferase reporter activity and ATP level in HEK293T-ACE2-TMPRSS2 cells challenged with VSV-G pseudotyped viruses and antidepressant-antipsychotic drugs. Comparison of luciferase reporter activity in HEK cells infected with SARS-CoV-2 spike- and VSV-G-pseudotyped viruses. 24 h treatment with **(a)** fluoxetine (n= 6-15), **(c)** clomipramine (n= 6-17), **(e)** chlorpromazine (n= 6-11), **(g)** flupenthixol (n= 6-17), **(i)** pimozide (n= 5-15) and **(k)** reboxetine (n= 11-14) reduced the luciferase reporter activity (blue dashed line). Decrease of ATP level was observed following 24 h treatment with **(a)** fluoxetine (n= 4-8), **(c)** clomipramine (n= 4-8), **(e)** chlorpromazine (n= 4-8), **(i)** pimozide (n= 4-8), and **(k)** reboxetine (n= 4-8) indicating the reduction of cell viability in infected cells (orange dashed line or full circles). **(g)** Flupenthixol did not alter the viability of cells (orange full circles, n= 4-8). Treatment with **(b)** fluoxetine (5, 10, 20 μM), **(d)** clomipramine (1, 5, 10 μM), **(f)** chlorpromazine (1 and 5 μM), **(h)** flupenthixol (1, 3, 5 μM), **(j)** pimozide (5 μM), and **(l)** reboxetine (20 and 50 μM) were more effective against the infection by SARS-CoV-2 spike pseudotyped viruses (red dashed line) than VSV-G pseudotyped viruses (blue dashed line). #$p < 0.05$ from control group (0), *$p < 0.05$ SARS-CoV-2 vs. VSV-G. Data represented as mean ± SEM.

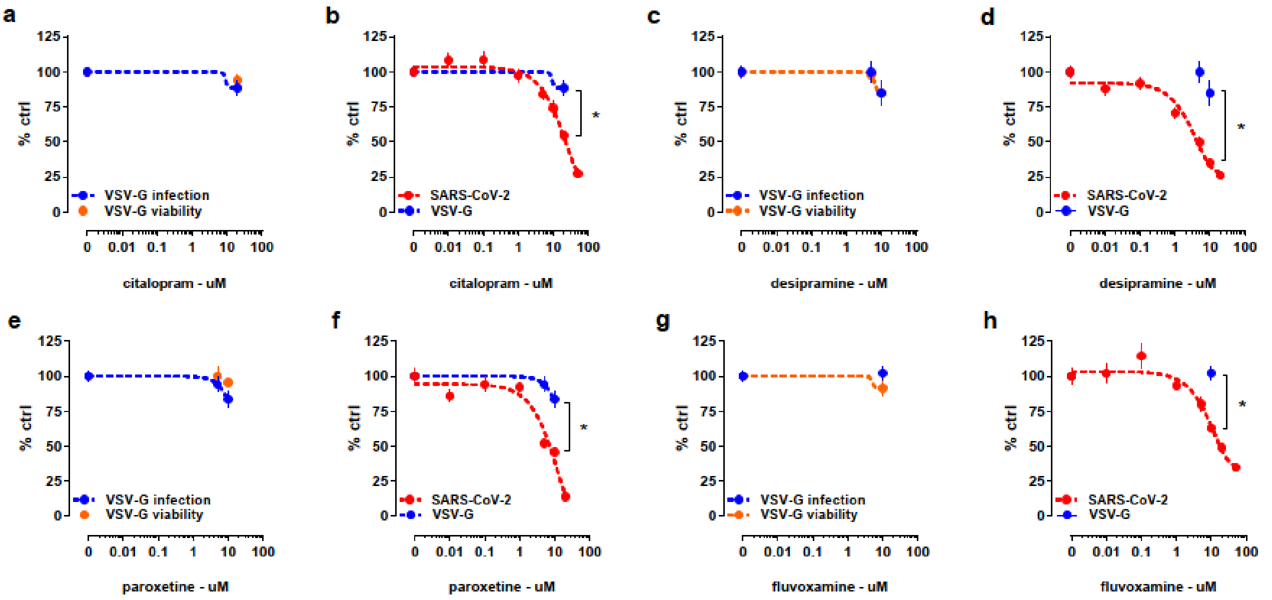

Supplementary Figure 7. Luciferase reporter activity and ATP level in HEK293T-ACE2-TMPRSS2 cells challenged with VSV-G pseudotyped viruses and antidepressant-antipsychotic drugs. Comparison of luciferase reporter activity in HEK cells infected with SARS-CoV-2 spike- and VSV-G-pseudotyped viruses. 24 h treatment with **(a)** citalopram (n= 11), **(c)** desipramine (n= 11), **(e)** paroxetine (n= 11), and **(g)** fluvoxamine (n= 11), did not significantly alter the luciferase reporter activity (blue dashed line or full circles). No change was observed in ATP level after treatment with **(a)** citalopram (n= 4-8), **(c)** desipramine (n= 4-8), **(e)** paroxetine (n= 4-8), and **(g)** fluvoxamine (n= 4-8) indicating unaltered cell viability in infected cells (orange dashed line or full circles). Treatment with **(b)** citalopram (20 μM), **(d)** desipramine (5 and 10 μM), **(f)** paroxetine (5 and 10 μM), and **(h)** fluvoxamine (10 μM) were more effective against the infection by SARS-CoV-2 spike pseudotyped viruses (red dashed line) than VSV-G pseudotyped viruses (blue dashed line or full circle). *$p < 0.05$ SARS-CoV-2 vs. VSV-G. Data represented as mean ± SEM.

2.2 Supplementary Tables

Supplementary Table 1. Human primers used for qPCR

**Primer Sequence (5' > 3')**	**Gene name**	**Amplicon Size (bp)**	**Reference**
F: GGGATCAGAGATCGGAAGAAGAAA<br>R: AGGAGGTCTGAACATCATCAGTG	*ACE2* (human) pair 1	124	(Ma et al., 2020)
F: AAACATACTGTGACCCCGCAT<br>R: CCAAGCCTCAGCATATTGAACA	*ACE2* (human) pair 2	199	(Ma et al., 2020)
F: AATCGGTGTGTTCGCCTCTAC<br>R: CGTAGTTCTCGTTCCAGTCGT	*TMPRSS2* (human) pair 1	106	(Ma et al., 2020)
F: CACTGTGCATCACCTTGACC<br>R: ACACACCGATTCTCGTCCTC	*TMPRSS2* (human) pair 2	196	(Esumi et al., 2015)
F: GAGTCAACGGATTTGGTCGT<br>R: GACAAGCTTCCCGTTCTCAG	*GAPDH* (human)	185	(Yin et al., 2020)
F: CCTTCTTCCGTGGGGTTAG<br>R: GCAGTTGCAGCTGTCATGTT	*FURIN* (human)	98	(Yin et al., 2020)

Supplementary Table 2. Statistical analysis of the data.

Graph	Test
2A	Fluoxetine<br>One-way ANOVA:<br>Luciferase assay: Kruskal-Wallis= 107.3, p< 0.0001*<br>Cell viability: F(3,20)= 134.6, p< 0.0001*
2B	Citalopram<br>One-way ANOVA:<br>Luciferase assay: F(7,148)= 30.84, p< 0.0001*<br>Cell viability: F (3,20)= 23.11, p< 0.0001*
2C	Paroxetine<br>One-way ANOVA:<br>Luciferase assay: Kruskal-Wallis = 65.37, p< 0.0001*<br>Cell viability: F(3,16)= 98.75, p< 0.0001*
2D	Fluvoxamine<br>One-way ANOVA:<br>Luciferase assay: Kruskal-Wallis = 91.16, p< 0.0001*<br>Cell viability: F(4,25)= 8.047, p= 0.0003*
2E	Venlafaxine<br>One-way ANOVA:<br>Luciferase assay: F(7,87)= 24.08, p< 0.0001*<br>Cell viability: F(4,20)= 1.058, p= 0.4030
2F	Reboxetine<br>One-way ANOVA:<br>Luciferase assay: F(6,77)= 14.30, p< 0.0001*<br>Cell viability: F(4,25)= 1.377, p= 0.2702
2G	Clomipramine<br>One-way ANOVA:<br>Luciferase assay: Kruskal-Wallis= 81.31, p< 0.0001*<br>Cell viability: F(3,20)= 16.16, p< 0.0001*
2H	Imipramine<br>One-way ANOVA:<br>Luciferase assay: Kruskal-Wallis= 75.25, p< 0.0001*<br>Cell viability: Kruskal-Wallis= 26.58, p< 0.0001*
2I	Desipramine<br>One-way ANOVA:<br>Luciferase assay: Kruskal-Wallis = 66.85, p< 0.0001*<br>Cell viability: F(3,16)= 16.48, p< 0.0001*
3A	Fluoxetine<br>Two-way ANOVA<br>Interaction: F(3,40)= 116.2, p< 0.0001*<br>Time: F(1,40)= 181.7, p< 0.0001*<br>Drug: F(3,40)= 116.2, p< 0.0001*
3B	Fluoxetine<br>One-way ANOVA:<br>Kruskal-Wallis= 19.49, p= 0.0002*
3C	Fluoxetine<br>One-way ANOVA:<br>F(3,20)= 65.53, p< 0.0001*
3D	Citalopram<br>Two-way ANOVA<br>Interaction: F(3,38)= 9.898, p< 0.0001*<br>Time: F(1,38)= 117.6, p< 0.0001*<br>Drug: F(3,38)= 9.903, p< 0.0001*
3E	Citalopram<br>One-way ANOVA:<br>Kruskal-Wallis= 9.689, p= 0.0214*
3F	Citalopram<br>One-way ANOVA:<br>F(3,20)= 11.87, p= 0.0001*
3G	Reboxetine<br>Two-way ANOVA<br>Interaction: F(3,40)= 3.448, p= 0.0254*<br>Time: F(1,40)= 151.9, p< 0.0001*<br>Drug: F(3,40)= 3.446, p= 0.0255*
3H	Reboxetine<br>One-way ANOVA:<br>Kruskal-Wallis= 9.738, p= 0.0209*

3I	Reboxetine<br>One-way ANOVA:<br>F(3,20)= 3.974, p= 0.0226*
3J	Clomipramine<br>Two-way ANOVA<br>Interaction: F(3,40)= 34.23, p< 0.0001*<br>Time: F(1,40)= 54.43, p< 0.0001*<br>Drug: F(3,40)= 34.22, p< 0.0001*
3K	Clomipramine<br>One-way ANOVA:<br>Kruskal-Wallis= 15.08, p= 0.0017*
3L	Clomipramine<br>One-way ANOVA:<br>F(3,20)= 68.07, p< 0.0001*
3M	Imipramine<br>Two-way ANOVA<br>Interaction: F(2,30)= 31.85, p< 0.0001*<br>Time: F(1,30)= 106.5, p< 0.0001*<br>Drug: F(2,30)= 31.85, p< 0.0001*
3N	Imipramine<br>One-way ANOVA:<br>F(2,15)= 2.498, p= 0.1158
3O	Imipramine<br>One-way ANOVA:<br>F(2,15)= 2.551, p= 0.1113
3P	Venlafaxine<br>Two-way ANOVA<br>Interaction: F(3,39)= 0.7408, p= 0.5342<br>Time: F(1,39)= 55.65, p< 0.0001*<br>Drug: F(3,39)= 0.7512, p= 0.5283
3Q	Venlafaxine<br>One-way ANOVA:<br>Kruskal-Wallis= 2.947, p= 0.3999
3R	Venlafaxine<br>One-way ANOVA:<br>F(3,20)= 6.286, p= 0.0035*
4A	Chlorpromazine<br>One-way ANOVA:<br>Luciferase assay: Kruskal-Wallis= 45.21, p< 0.0001*<br>Cell viability: F(2,21)= 42.42, p< 0.0001*
4B	Flupenthixol<br>One-way ANOVA:<br>Luciferase assay: F(6,77)= 149.3, p< 0.0001*<br>Cell viability: F(3,16)= 19.04, p< 0.0001*
4C	Pimozide<br>One-way ANOVA:<br>Luciferase assay: Kruskal-Wallis= 50.77, p< 0.0001*<br>Cell viability: F(3,16)= 77.59, p< 0.0001*
4D	Chlorpromazine<br>Two-way ANOVA<br>Interaction: F(3,38)= 19.98, p< 0.0001*<br>Time: F(1,38)= 113.3, p< 0.0001*<br>Drug: F(3,38)= 19.98, p< 0.0001*
4E	Chlorpromazine<br>One-way ANOVA:<br>Kruskal-Wallis= 3.152, p= 0.3688
4F	Chlorpromazine<br>One-way ANOVA:<br>F(3,20)= 18.48, p< 0.0001*
5A	Fluoxetine<br>Two-way ANOVA:<br>Treatment: F(1,98)= 894.2, p< 0.0001*<br>Mutant: F(2,98)= 1.481, p= 0.2324<br>Interaction: F(2,98)= 1.481, p= 0.2324
5B	Fluoxetine<br>Two-way ANOVA:<br>Treatment: F(1,30)= 116.4, p< 0.0001*<br>Mutant: F(1,30)= 0.5339, p= 0.4707<br>Interaction: F(1,30)= 0.5339, p= 0.4707
5C	Fluoxetine<br>Two-way ANOVA:

	Treatment: F(1,54)= 255.5, p< 0.0001*<br>Mutant: F(1,54)= 0.4988, p= 0.4831<br>Interaction: F(1,54)= 0.4988, p= 0.4831
5D	B.1.1.7 - Fluoxetine<br>Interaction: F(1,20)= 4.847, p= 0.0396*<br>Time: F(1,20)= 4.970, p= 0.0374*<br>Drug: F(1,20)= 4.845, p= 0.0396*
5E	B.1.1.7 - Fluoxetine<br>Unpaired t-test, Mann-Whitney<br>p= 0.0022*
5F	B.1.1.7 - Fluoxetine<br>Unpaired t-test<br>t(10)= 12.26, p< 0.0001*
5G	B.1.351 - Fluoxetine<br>Interaction: F(1,20)= 35.41, p< 0.0001*<br>Time: F(1,20)= 38.24, p< 0.0001*<br>Drug: F(1,20)= 35.41, p< 0.0001*
5H	B.1.351 - Fluoxetine<br>Unpaired t-test<br>t(10)= 3.984, p= 0.0026*
5I	B.1.351 - Fluoxetine<br>Unpaired t-test<br>t(10)= 5.777, p=0.0002*
5J	B.1.617.2 - Fluoxetine<br>Interaction: F(1,20)= 300.3, p< 0.0001*<br>Time: F(1,20)= 380.7, p< 0.0001*<br>Drug: F(1,20)= 306.7, p< 0.0001*
5K	B.1.617.2 - Fluoxetine<br>Unpaired t-test, Mann-Whitney<br>p= 0.0022*
5L	B.1.617.2 - Fluoxetine<br>Unpaired t-test<br>t(10)= 8.975, p< 0.0001*
S2	Camostat mesylate<br>One-way ANOVA:<br>Kruskal-Wallis= 31.58, p< 0.0001*
S3a	Fluoxetine<br>Two-way ANOVA:<br>Interaction: F(3,40)= 0.3057, p= 0.8211<br>Drug: F(3,40)= 39.28, p< 0.0001*<br>Infection: F(1,40)= 0.004110, p= 0.9492
S3b	Citalopram<br>Two-way ANOVA:<br>Interaction: F(3,40)= 0.3920, p = 0.7594<br>Drug: F(3,40)= 19.11, p< 0.0001*<br>Infection: F(1,40)= 2.759, p= 0.1045
S3c	Paroxetine<br>Two-way ANOVA:<br>Interaction: F(3,40)= 1.662, p= 0.1904<br>Drug: F(3,40)= 187.4, p< 0.0001*<br>Infection: F(1,40)= 0.7991, p= 0.3767
S3d	Fluvoxamine<br>Two-way ANOVA:<br>Interaction: F(4,50)= 4.186, p= 0.0053*<br>Drug: F(4,50)= 26.12, p< 0.0001*<br>Infection: F(1,50)= 0.1847, p= 0.6692
S3e	Venlafaxine<br>Two-way ANOVA:<br>Interaction: F(4,50)= 2.216, p= 0.0806<br>Drug: F(4,50)= 4.099, p= 0.0060*<br>Infection: F(1,50)= 4.263, p= 0.0442*
S3f	Reboxetine<br>Two-way ANOVA:<br>Interaction: F(4,50) = 0.2360, p= 0.9167<br>Drug: F(4,50)= 1.152, p= 0.3431<br>Infection: F(1,50)= 0.05549, p= 0.8147
S3g	Clomipramine<br>Two-way ANOVA:<br>Interaction: F(3,40)= 1.566, p= 0.2126<br>Drug: F(3,40)= 140.3, p< 0.0001*

	Infection: F(1,40)= 0.1518, p= 0.6989
S3h	Imipramine<br>Two-way ANOVA:<br>Interaction: F(4,50) = 0.8544, p= 0.4977<br>Drug: F(4,50)= 17.74, p< 0.0001*<br>Infection: F(1,50)= 1.284, p= 0.2625
S3i	Desipramine<br>Two-way ANOVA:<br>Interaction: F(3,40)= 1.182, p= 0.3287<br>Drug: F(3,40) = 44.60, p< 0.0001*<br>Infection: F(1,40)= 0.06867, p= 0.7946
S3j	Chlorpromazine<br>Two-way ANOVA:<br>Interaction: F(2,30)= 0.1997, p= 0.8200<br>Drug: F(2,30)= 21.61, p< 0.0001*<br>Infection: F(1,30)= 0.02015, p= 0.8881
S3k	Flupenthixol<br>Two-way ANOVA:<br>Interaction: F(3,40)= 1.230, p= 0.3113<br>Drug: F(3,40)= 14.44, p< 0.0001*<br>Infection: F(1,40)= 10.19, p= 0.0027*
S3l	Pimozide<br>Two-way ANOVA:<br>Interaction: F(3,40)= 3.032, p= 0.0403*<br>Drug: F(3,40)= 72.62, p< 0.0001*<br>Infection: F(1,40)= 3.851, p= 0.0567
S4a	Two-way ANOVA<br>Interaction: F(3,37)= 0.5979, p= 0.6204<br>Treatment: F(3,37)= 3.821, p= 0.0176*<br>Infection: F(1,37)= 0.9481, p= 0.3365
S4b	Two-way ANOVA<br>Interaction: F(3,37)= 0.3392, p= 0.7971<br>Treatment: F(3,37)= 16.29, p< 0.0001*<br>Infection: F(1,37)= 0.4117, p= 0.5251
S4d	ACE2<br>Two-way ANOVA:<br>Interaction: F(1,44)= 0.1690, p= 0.6830<br>Infection: F(1,44)= 0.03670, p= 0.8490<br>Fluoxetine: F(1,44)= 0.4414, p= 0.5099
S4e	TMPRSS2<br>Two-way ANOVA:<br>Interaction: F(1,44)= 0.8336, p= 0.3662<br>Infection: F(1,44)= 0.1126, p= 0.7388<br>Fluoxetine: F(1,44)= 12.96, p= 0.0008*
S5a	Ketamine<br>One-way ANOVA:<br>Luciferase assay: F(7,156)= 1.654, p= 0.1243
S5b	2R,6R-HNK<br>One-way ANOVA:<br>Luciferase assay: Kruskal-Wallis= 5.784, p= 0.5652
S5c	2S,6S-HNK<br>One-way ANOVA:<br>Luciferase assay: F(7,111)= 1.439, p= 0.1970
S5d	Rolipram<br>One-way ANOVA:<br>Luciferase assay: F(7,51)= 0.4432, p= 0.8702
S5e	Phenelzine<br>One-way ANOVA:<br>Luciferase assay: Kruskal-Wallis= 14.62, p= 0.0412
S5f	Carbamazepine<br>One-way ANOVA:<br>Luciferase assay: F(6,35)= 2.325, p= 0.0540
S6a	Fluoxetine:<br>One-way ANOVA:<br>Luciferase: F(3,39)= 6.634, p= 0.0010*<br>Cell viability: F(4,19)= 3.920, p= 0.0174*
S6b	Fluoxetine:<br>Two-way ANOVA:<br>Treatment: F(3,125)= 39.26, p< 0.0001*<br>Virus: F(1,125)= 56.79, p< 0.0001*<br>Interaction: F(3,125)= 8.490, p< 0.0001*

S6c	Clomipramine<br>One-way ANOVA:<br>Luciferase: $F(5,51)= 4.259$, $p= 0.0026$*<br>Cell viability: $F(3,16)= 9.611$, $p= 0.0007$*
S6d	Clomipramine<br>Two-way ANOVA:<br>Treatment: $F(5,146)= 39.23$, $p< 0.0001$*<br>Virus: $F(1,146)= 57.05$, $p< 0.0001$*<br>Interaction: $F(5,146)= 16.59$, $p< 0.0001$*
S6e	Chlorpromazine<br>One-way ANOVA:<br>Luciferase: $F(3,34)= 9.231$, $p= 0.0001$*<br>Cell viability: $F(2,13)= 10.42$, $p= 0.0020$*
S6f	Chlorpromazine<br>Two-way ANOVA:<br>Treatment: $F(3,86)= 38.78$, $p< 0.0001$*<br>Virus: $F(1,86)= 42.66$, $p< 0.0001$*<br>Interaction: $F(3,86)= 9.855$, $p< 0.0001$*
S6g	Flupenthixol<br>One-way ANOVA:<br>Luciferase: $F(3,36)= 5.700$, $p= 0.0027$*<br>Cell viability: $F(3,16)= 0.3592$, $p= 0.7833$
S6h	Flupenthixol<br>Two-way ANOVA:<br>Treatment: $F(3,80)= 59.09$, $p< 0.0001$*<br>Virus: $F(1,80)= 66.26$, $p< 0.0001$*<br>Interaction: $F(3,80)= 12.42$, $p< 0.0001$*
S6i	Pimozide<br>One-way ANOVA:<br>Luciferase: $F(2,27)= 5.234$, $p= 0.0120$*<br>Cell viability: $F(2,13)= 5.578$, $p= 0.0178$*
S6j	Pimozide<br>Two-way ANOVA:<br>Treatment: $F(2,60)= 34.24$, $p< 0.0001$*<br>Virus: $F(1,60)= 4.542$, $p= 0.0372$*<br>Interaction: $F(2,60)= 2.295$, $p= 0.1096$
S6k	Reboxetine<br>One-way ANOVA:<br>Luciferase: $F(2,36)= 4.344$, $p= 0.0204$*<br>Cell viability: $F(2,13)= 14.67$, $p= 0.0005$*
S6l	Reboxetine<br>Two-way ANOVA:<br>Treatment: $F(2,69)= 26.49$, $p< 0.0001$*<br>Virus: $F(1,69)= 16.47$, $p= 0.0001$*<br>Interaction: $F(2,69)= 5.190$, $p= 0.0079$*
S7a	Citalopram<br>Unpaired t-test:<br>Luciferase: $t(20)= 1.612$, $p= 0.1227$<br>Cell viability: $t(10)= 1.017$, $p= 0.3333$
S7b	Citalopram<br>Two-way ANOVA:<br>Treatment: $F(1,58)= 36.77$, $p< 0.0001$*<br>Virus: $F(1,58)= 12.95$, $p= 0.0007$*<br>Interaction: $F(1,58)= 12.95$, $p= 0.0007$*
S7c	Desipramine<br>One-way ANOVA:<br>Luciferase: $F(2,30)= 1.396$, $p= 0.2633$<br>Cell viability: $F(2,13)= 3.591$, $p= 0.0573$
S7d	Desipramine<br>Two-way ANOVA:<br>Treatment: $F(2,63)= 24.24$, $p< 0.0001$*<br>Virus: $F(1,63)= 49.05$, $p< 0.0001$*<br>Interaction: $F(2,63)= 12.26$, $p< 0.0001$*
S7e	Paroxetine<br>One-way ANOVA:<br>Luciferase: $F(2,30)= 2.296$, $p= 0.1181$<br>Cell viability: $F(2,13)= 0.2985$, $p= 0.7469$
S7f	Paroxetine<br>Two-way ANOVA:<br>Treatment: $F(2,63)= 28.49$, $p< 0.0001$*

	Virus: $F(1,63)= 44.34$, $p< 0.0001$*<br>Interaction: $F(2,63)= 11.19$, $p< 0.0001$*
S7g	Fluvoxamine<br>Unpaired t-test:<br>Luciferase: $t(20)= 0.3183$, $p= 0.7536$<br>Cell viability: $t(10)= 1.378$, $p= 0.1983$
S7h	Fluvoxamine<br>Two-way ANOVA:<br>Treatment: $F(1,56)= 8.557$, $p= 0.0050$*<br>Virus: $F(1,56)= 10.78$, $p= 0.0018$*<br>Interaction: $F(1,56)= 10.78$, $p= 0.0018$*

Supplementary Table 3. IC_{50}, CC_{50} and selectivity index (SI) of compounds that were tested in pseudovirus-infected HEK-ACE2-TMPRSS2 cells.

**Compound**	**IC_{50} (µM)**	**CC_{50} (µM)**	**SI**
Fluoxetine	5.992	41.48	6.92
Citalopram	27.51	274.4	9.97
Paroxetine	12.55	22.10	1.76
Fluvoxamine	10.54	75.26	7.14
Venlafaxine	36.35	No fitting	No fitting
Reboxetine	17.69	No fitting	No fitting
Clomipramine	0.7517	65.65	87.34
Imipramine	3.006	68.63	22.83
Desipramine	8.097	68.86	8.50
Chlorpromazine	0.9725	15.86	16.31
Flupenthixol	1.072	19.41	18.11
Pimozide	4.539	15.01	3.31

2.3 Supplement References

Esumi, M., Ishibashi, M., Yamaguchi, H., Nakajima, S., Tai, Y., Kikuta, S., Sugitani, M., Takayama, T., Tahara, M., Takeda, M., & Wakita, T. (2015). Transmembrane serine protease TMPRSS2 activates hepatitis C virus infection. *Hepatology*, *61*(2), 437–446. https://doi.org/10.1002/hep.27426

Ma, D., Chen, C.-B., Jhanji, V., Xu, C., Yuan, X.-L., Liang, J.-J., Huang, Y., Cen, L.-P., & Ng, T. K. (2020). Expression of SARS-CoV-2 receptor ACE2 and TMPRSS2 in human primary conjunctival and pterygium cell lines and in mouse cornea. *Eye*, 1–8. https://doi.org/10.1038/s41433-020-0939-4

Yin, J., Chang, H.-M., Yi, Y., Yao, Y., & Leung, P. C. K. (2020). TGF-β1 Increases GDNF Production by Upregulating the Expression of GDNF and Furin in Human Granulosa-Lutein Cells. *Cells*, *9*(1). https://doi.org/10.3390/cells9010185
